# A New Snake Skull from the Paleocene of Bolivia Sheds Light on the Evolution of Macrostomatans

**DOI:** 10.1371/journal.pone.0057583

**Published:** 2013-03-01

**Authors:** Agustín Scanferla, Hussam Zaher, Fernando E. Novas, Christian de Muizon, Ricardo Céspedes

**Affiliations:** 1 Consejo Nacional de Investigaciones Científicas Y Técnicas, Instituto de Bio y Geociencias del NOA, Museo de Ciencias Naturales de Salta, Salta, Argentina; 2 Museu de Zoologia, Universidade de São Paulo, São Paulo, Brasil; 3 Laboratorio de Anatomía Comparada y Evolución de los Vertebrados, Museo Argentino de Ciencias Naturales “Bernardino Rivadavia”, Buenos Aires, Argentina; 4 UMR 7207 CNRS, CP 38, Département Histoire de la Terre, Paris, France; 5 Museo de Historia Natural “Alcide D´Orbigny”, Cochabamba, Bolivia; Ludwig-Maximilians-Universität München, Germany

## Abstract

Macrostomatan snakes, one of the most diverse extant clades of squamates, display an impressive arsenal of cranial features to consume a vast array of preys. In the absence of indisputable fossil representatives of this clade with well-preserved skulls, the mode and timing of these extraordinary morphological novelties remain obscure. Here, we report the discovery of *Kataria anisodonta* n. gen. n. sp., a macrostomatan snake recovered in the Early Palaeocene locality of Tiupampa, Bolivia. The holotype consists of a partial, minute skull that exhibits a combination of booid and caenophidian characters, being the presence of an anisodont dentition and diastema in the maxilla the most distinctive trait. Phylogenetic analysis places *Kataria* basal to the Caenophidia+Tropidophiidae, and represents along with bolyeriids a distinctive clade of derived macrostomatans. The discovery of *Kataria* highlights the morphological diversity in the maxilla among derived macrostomatans, demonstrating the relevance of maxillary transformations in the evolution of this clade. *Kataria* represents the oldest macrostomatan skull recovered, revealing that the diversification of macrostomatans was well under way in early Tertiary times. This record also reinforces the importance of Gondwanan territories in the history of snakes, not only in the origin of the entire group but also in the evolution of ingroup clades.

## Introduction

In recent years, the discovery of new and nearly complete fossil specimens as well as the reanalysis of previously known materials has dramatically improved our knowledge about the evolution of snakes [1], [2], [3], [4], [5], [6], [7], [8]. Such renewed interest on ophidian evolution is based primarily on the study of fossil exemplars that preserved skull elements, an unusual situation for this group of squamates. However, their phylogenetic position fails to offer any clue about the evolution of the more advanced extant snake clades.

Macrostomata constitutes the most diverse group of snakes today, including nearly all of the extant species [9]. This clade is characterized by a suite of exceptionally distinct skull traits responsible for an increased gape size that permits the ingestion of large preys [10], [11], [12]. Despite these numerous uniquely derived features, recent molecular studies [13], [14] suggested that this diverse group constitutes a polyphyletic lineage, with tropidophiids clustering outside macrostomatans as the sister-group of *Anilius*, whereas remaining macrostomatans form a monophyletic unit that includes the rest of “anilioid” alethinophidians. These phylogenetic hypotheses suggest that macrostomatan traits might have been lost or appeared independently twice within alethinophidian snakes. However, the unstable position of uropeltids (including *Anomochilus* and *Cylindrophis*) within macrostomatan snakes, shown in several independent molecular phylogenies, precludes the establishment of a clear evolutionary scenario and suggests that additional evidence is needed to clarify this issue. Meanwhile, we prefer to consider here a monophyletic Macrostomata, as suggested by all previous morphological analyses [2], [4], [5], [6], [7], and corroborated by the most exhaustive morphology-based phylogeny of Squamata [15]. According to these preferred phylogenetic proposals, Macrostomata includes the basal forms Xenopeltidae and Loxocemidae, and two more derived subclades that display distinct morphologies and ecological requirements: 1) a group formed by the ungaliophiines, erycines, pythonines, and boines; and 2) a group including bolyeriids, tropidophiids, and caenophidians (acrochordids and colubroideans). Despite their current impressive diversity and cosmopolitan distribution, nearly all that we know about the evolution of the macrostomatan cranial bauplan comes from studies focused in recent forms [11], [16], [17]. Indeed, due to the lack of well-preserved cranial fossil remains, little is known about the origin and diversification of their highly specialized cranial features.

Here we report a new fossil snake from Paleocene beds of Bolivia that emerges in our analysis as the sister-group of the clade formed by bolyeriids, tropidophiids, and caenophidians. The new fossil preserves the most complete and oldest macrostomatan skull found so far, filling an important gap in the evolutionary history of this relevant clade of snakes. It further provides a relevant calibration point to discuss the evolutionary timing of advanced terms of macrostomatan snakes.

## Methods

All necessary permits were obtained for the described study from Comité de Preservación del Patrimonio Departamental (Cochabamba department, Bolivia), which complied with all relevant regulations.

### Phylogenetic Analysis

The character-taxon matrix used in the phylogenetic analysis is mainly based on a published phylogenetic analysis [7], with the addition of *Kataria* and 2 new characters. Thus, the reported analysis results in a data matrix of 156 characters scored across 23 taxa (Text S1, Dataset S1). All characters were treated as unordered, as in the original phylogenetic analysis [7].

We analysed our dataset using TNT [18], [19] with a heuristic search of 1000 replicates of Wagner trees followed by tree bisection-reconnection (TBR) branch swapping. All characters were equally weighted. Zero length branches were collapsed if they lack support under any of the most parsimonious reconstructions. Also, two alternative support measures (Bremer support [20] and bootstrap resampling) were used to evaluate the robustness of the nodes of the obtained most parsimonious trees (see Text S1).

Additionally, the morphological dataset was analyzed with the 13 extant terminal taxa constrained with a backbone formed by the topology derived from the molecular analysis performed by Wiens and colleagues [21]. This analysis served to test the effect of a molecular topology on our dataset and, more specifically, on the phylogenetic position of *Kataria*. The nine fossil taxa in the dataset were pruned (using “pruntax” command in TNT) and allowed to insert freely into their optimal positions during the constrained analysis. The molecular tree was constructed using the “edit” command in TNT. The constrained analysis was performed using a heuristic tree search with the molecular tree enforced as a backbone with “force” and “cons” commands in TNT.

### Nomenclatural Acts

The electronic edition of this article conforms to the requirements of the amended International Code of Zoological Nomenclature, and hence the new names contained herein are available under that Code from the electronic edition of this article. This published work and the nomenclatural acts it contains have been registered in ZooBank, the online registration system for the ICZN. The ZooBank LSIDs (Life Science Identifiers) can be resolved and the associated information viewed through any standard web browser by appending the LSID to the prefix “http://zoobank.org/”. The LSID for this publication is: urn:lsid:zoobank.org:pub:30F0320E-11D7-450A-B108-24EA5D56827C. The electronic edition of this work was published in a journal with an ISSN, and has been archived and is available from the following digital repositories: PubMed Central, LOCKSS.

## Results

### Systematic Paleontology

Serpentes Linnaeus 1758.

Alethinophidia Nopcsa 1923.

Macrostomata Müller 1831.

*Kataria anisodonta* Scanferla, Zaher, Novas, Muizon and Céspedes sp. nov. urn:lsid:zoobank.org:act:EEF2F3CF-6CBA-447A-953B-7539F3C90388.

#### Etymology

The generic name derives from the Aymara word “*Katari*”, a winged mythological snake of South American Andean people. The specific name refers to the particular maxillary dentition, combining the Greek words “*aniso”* (heterogeneous) and “*donta”* (tooth).

#### Holotype

**Figure 1:**
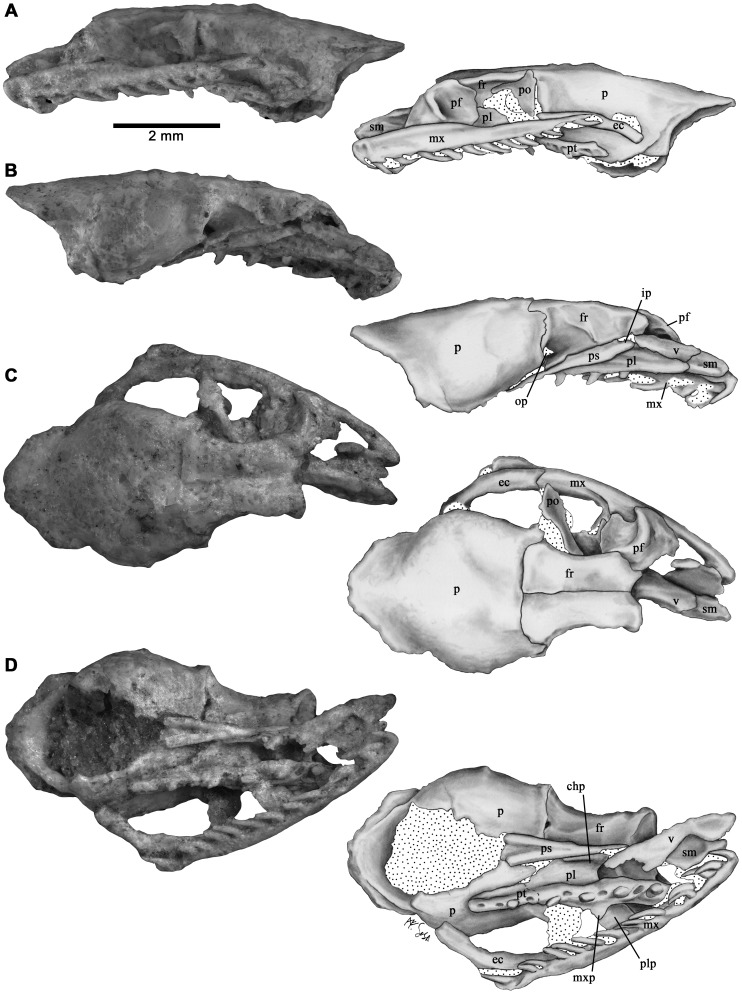
The skull of *Kataria anisodonta* (MHNC 13323). Photographs and half-tone drawings in (A) left lateral, (B) right lateral, (C) dorsal and (D) ventral views. Dotted areas indicate matrix. chp, choanal process; ec, ectopterygoid; fr, frontal; ip, interchoanal process; mx, maxilla; mxp, maxillary process; op, optic foramen; p, parietal; pf, prefrontal; pl, palatine; plp, palatine process; po, postorbital; ps, parasphenoid; pt, pterygoid; sm, septomaxilla; v, vomer.

MHNC 13323 (Museo de Historia Natural de Cochabamba “Alcides D´orbigny, Cochabamba, Bolivia), an articulated incomplete skull consisting of a left vomer, incomplete left septomaxilla, left maxilla, left ectopterygoid, left palatine, the anterior tip of the left pterygoid, left postorbital, both frontals, parietal, and parasphenoid rostrum (Fig. 1).

#### Locality and horizon

Tiupampa locality, Mizque province of the department of Cochabamba, Bolivia. Medium-grained sandstones of the middle levels of Santa Lucía Formation, Early Paleocene (Danian [22]).

#### Diagnosis

A small, derived macrostomatan snake that can be distinguished from all other members of Serpentes by the following combination of characters: an elongated vomer with a reduced contribution to the vomeronasal fenestra; one foramen piercing the cavity housing Jacobson’s organ; maxilla with 21 tooth positions and the posterior most tooth separated by a diastema from the others; ectopterygoid with a small medial process and a ventral articular surface with the pterygoid; a broad choanal process of the palatine; optic fenestra formed by both frontal and parietal.

### Ontogenetic Stage of the Specimen

Estimation of the ontogenetic stage of a fossil snake skull is problematic because there are few studies on postnatal ontogeny in snakes. Those that do exist all focus on allometric variations in skull elements [23], [24], [25]. These works, however, do not provide strong grounds to assess the ontogenetic stage of *Kataria* because they are based on quantitative characters with no descriptions of useful discrete features.

At a first glance, the tiny size (Table 1) and poorly developed parietal table and sagittal crest appear to indicate that the holotypic specimen of *Kataria* represents a juvenile postnatal ontogenetic stage. However, adult specimens of several small-sized macrostomatan taxa (e.g. *Lichanura trivirgata*, *Apostolepis* spp.), approach *Kataria* in their size and parietal morphology. Hence, somatically mature small-bodied snakes exhibit skull morphology similar to juveniles of larger taxa.

**Table 1:** Selected measurements of *Kataria anisodonta.*

Maxilla length	4,6
Ectopterygoid length	2,2
Palatine length	3,1*
Frontal length	1,75
Postorbital height	0,9

Measurements are in mm;

* refers to estimated value.

Based on personal observations, ontogenetic transformations in the postorbital bone may be useful in distinguishing ontogenetic stages in macrostomatan snakes. Like those of several adult macrostomatan skulls examined (see Text S1), the postorbital of *Kataria* displays a distinct thickening of the postorbital shaft, and the posterior process of the dorsal head is enlarged. We consider that these anatomical traits present in the postorbital bone, together with the advanced state of ossification observed in this minute skull, suggest an adult postnatal ontogenetic stage for the holotype.

### Description and Comparisons

The type specimen consists of a small articulated skull, with some elements (e.g. snout bones) barely displaced. Its anatomy reveals booid traits in combination with some apomorphic features present in tropidophiids and caenophidian snakes.

**Figure 2:**
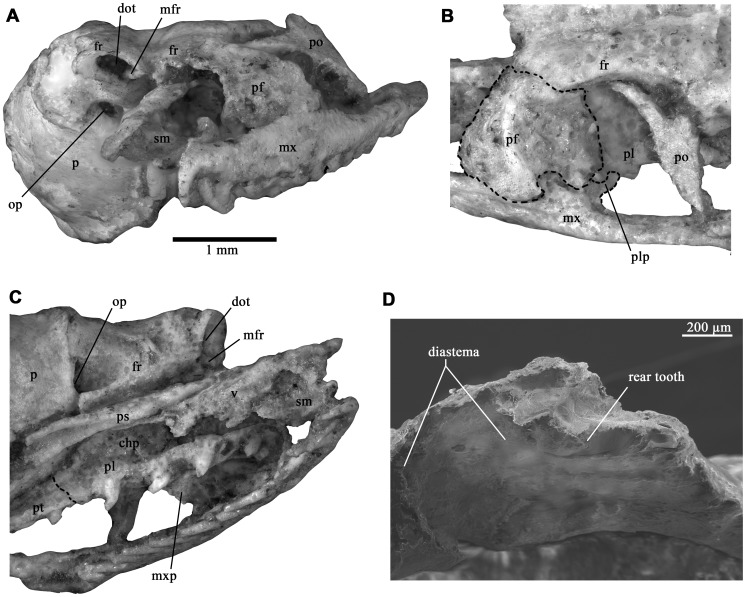
Details of the holotype specimen of *Kataria anisodonta*. (A) frontal view of the partial skull; (B) dorsolateral view of the left orbit; (C) ventral view of the palatal region; (D) scanning electron microscope image of the rear maxillary region. chp, choanal process; dot, ductus for olfactory tract; ec, ectopterygoid; fr, frontal; ip, interchoanal process; mfr, medial frontal flange; mx, maxilla; mxp, maxillary process; op, optic foramen; p, parietal; pf, prefrontal; pl, palatine; plp, palatine process; po, postorbital; ps, parasphenoid; pt, pterygoid; sm, septomaxilla; v, vomer.

The preserved snout bones of *Kataria* retain a typical “booid” morphology. The vomer is remarkably long, with well-developed vertical and horizontal posterior laminae (Fig. 1B), condition present in *Anilius* and many macrostomatans except caenophidians [26]. A single foramen pierces the posterior wall of the cavity housing Jacobson’s organ, and the sidewall of this structure is formed largely by the septomaxilla rather than the vomer, a condition shared also with non-caenophidian snakes [26]. Contrary to the plesiomorphic condition present in basal macrostomatans, the vomeronasal cupola of *Kataria* is closed medially by an extensive medial contact between vomer and septomaxilla (Fig. 1B). The lateral flange of the septomaxilla is present, but somewhat crushed, and shows a broad base. The septomaxilla projects caudally to the posterior border of the vomer to form a postero-dorsally ascending flange that reaches the frontal medially. The posterior tip of the ascending flange of the septomaxilla is clearly rounded, and there are no traces of articular structures that link this bone with the frontal subolfactory process. A slight dorsal displacement of the snout complex gives the impression of a septomaxilla-frontal contact (Fig. 2). However, suspension of the snout unit probably occurred through soft tissues and/or the nasal bone (not preserved in *Kataria*) instead of the septomaxilla.

**Figure 3:**
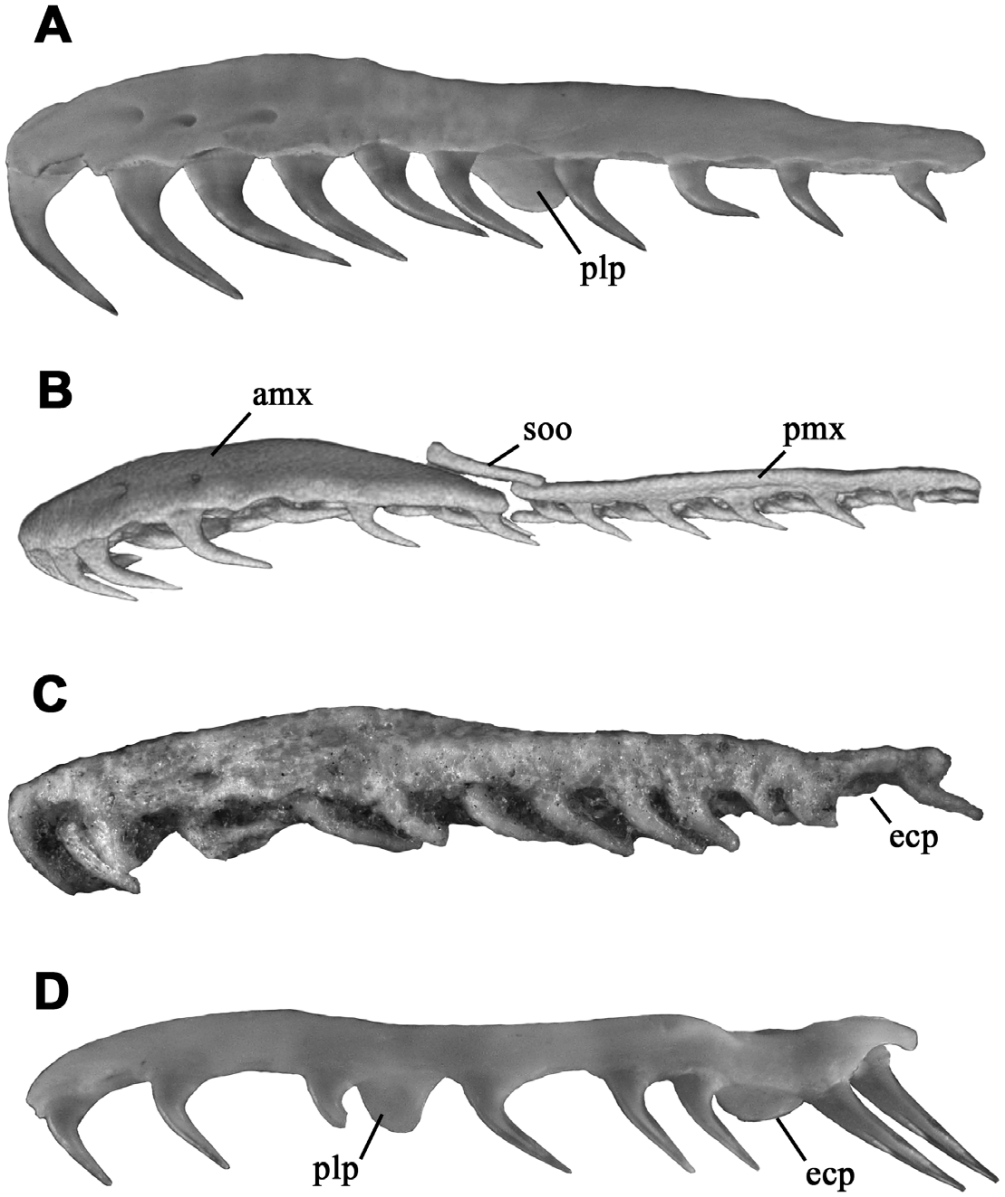
Lateral view of maxillary bones showing differences in tooth row morphology of macrostomatan snakes. (A) the boid *Eunectes notaeus*, (**b**) the bolyeriid *Casarea dussumieri* [44], (C) *Kataria anisodonta* and (D) the opistoglyphous colubroid *Philodryas trilineatus*. Not to scale. amx, anterior maxilla; ecp, ectopterygoid process; plp, palatine process; pmx, posterior maxilla; soo, suborbital ossification.

The maxilla of *Kataria* is the element of the palatomaxillary apparatus that displays most apomorphic and intriguing traits. This bone is elongated (Table 1) and has a slightly recurved shape as in many colubroideans (Fig. 3C), differing from the condition present in other macrostomatans where the anterior third of the maxilla curves medially. The rounded anterior tip indicates no close association with the premaxilla, suggesting a loose, ligamentous connection between these elements. As in tropidophiids and caenophidians, the lateral surface lacks the lateral foramina present in many booids. The medial (palatine) process is situated in the middle region, between the 9^th^ and 12^th^ tooth positions. Although the prefrontal and palatine bones obscure part of its morphology, it is possible to verify the absence of the foramen for the passage of the maxillary branch of the trigeminal nerve, a derived condition shared with caenophidian snakes. The ectopterygoid process is weakly expressed as a small ventromedial projection, located at the level of the space between the rear tooth and the rest of the tooth row. There are 21 tooth positions, distributed in two clearly defined sections (Fig. 3C). The anterior tooth row has 20 tooth positions occupied by elongate, needle-shaped recurved teeth. These teeth gradually diminish towards the posterior region, in contrast with the condition present in rear-fanged colubroids where teeth are similar in size. Notably, there is one tooth separated from the rest of the tooth row by a conspicuous, toothless gap. The maxilla in this toothless region is flat and ventro-dorsally thin with respect to the rest of the bone (Fig. 3C), and its dental (ventral) region is smooth and without traces of tooth sockets or interdental ridges [27]. A flexion can be seen in dorsal view starting just at the level of the last tooth. This trait is present in all opistoglyphous colubroids examined. The tip of the rear tooth is broken; however, the preserved portion is larger than the two teeth positioned just anterior to the toothless gap. The enamel surface of this tooth is not satisfactorily preserved, but it is possible to confirm that a groove is lacking (Fig. 2D). The left ectopterygoid is present and articulates with the maxilla (Fig. 1C–D). This bone bears a short shaft (Table 1), in constrast with the elongated shape present in tropidophiids and caenophidians. The anterior part, which has an angulated lateral margin, bears a small medial process similar to that present in many macrostomatan snakes. This region of the ectopterygoid overlaps the dorsomedial surface of the posterior tip of the maxilla. The caudal end of the ectopterygoid has a small, flattened region that articulates with the pterygoid when the latter is present. We here interpret that this region must have overlapped the pterygoid on its dorsal surface as in bolyeriids, tropidophiids and caenophidians. In macrostomatan snakes, the posterior region of the maxilla and the anterior head of the ectopterygoid form a horizontal overlapping joint. In *Kataria*, this articulation and the posterior articular surface of the ectopterygoid that contacts the pterygoid are in the same plane, supporting the dorsal ectopterygoid-pterygoid contact interpreted above. The general shape of the palatine bone resembles *Anilius* and tropidophiids, being characterized by a prominent dentigerous process, a broad-based choanal process, and a small laterally projected maxillary process pierced by a foramen that corresponds to the maxillary branch of trigeminal nerve (Fig. 1D). The ventral view reveals seven tooth positions, and preserved teeth are morphological similar to the marginal (maxillary) teeth. Unfortunately, the poorly preserved region of the palatine-pterygoid contact avoids any interpretation of the nature of this articulation. A small fragment corresponds to the anteriormost part of the palatine ramus of the pterygoid. This fragment retains four tooth positions and one of these reveals the base of a tooth. The tooth sockets and the preserved fragmentary tooth on the anterior fragment are smaller than the more posterior palatine tooth sockets, suggesting that the gradual transition in the size of palatal teeth known in many booids [28] was absent in *Kataria*.

The preserved left prefrontal of *Kataria* is in articulation with the frontal, but slightly rotated medially (Fig. 1A–C). As in boines, bolyeriids and tropidophiids, the lachrymal duct is open ventrally (Fig. 2B). Also, there is no indication of the typical boid dorsal lappet. On its lateral side, the prefrontal exhibits a short lateral lamina, and this bone retains only a posterior contact with the maxilla. Although the anteriormost region of the lateral lamina is incomplete, it is possible that this structure exhibited an anterior projection similar to the one present in *Casarea* [29]. The postorbital bone is well developed, being slightly displaced from its original position. The shape of the dorsal head resembles the forked condition present in tropidophiids, with the difference that its long anterior process was clearly in contact with the dorsolateral edge of the frontal bone. The longitude that shows this bone and the acute shape of the ventral tip indicate that the postorbital terminated well dorsal to the ectopterygoid-maxillary joint.

The frontals are slender bones that show complete (fused) interolfactory pillars (Fig. 2A), a condition shared with caenophidians [17]. In *Kataria*, as in the vast majority of macrostomatan snakes, the optic foramen lies between the frontal and parietal, excluding the parasphenoid rostrum. As in tropidophiids and caenophidians, the parietal of *Kataria* is longitudinally short, without the posterior supratemporal processes present in boid snakes. This bone bears small postorbital processes, clasped by the forked dorsal head of the postorbital. The overall shape of the preserved portion of the parasphenoid in *Kataria* is very similar to that of bolyeriids. This structure is elongate and slender, with concave ventral surface. Anteriorly, the parasphenoidal rostrum forms a ventrally projecting, narrow-based interchoanal process (Fig. 1B).

### Phylogenetic Analysis

**Figure 4:**
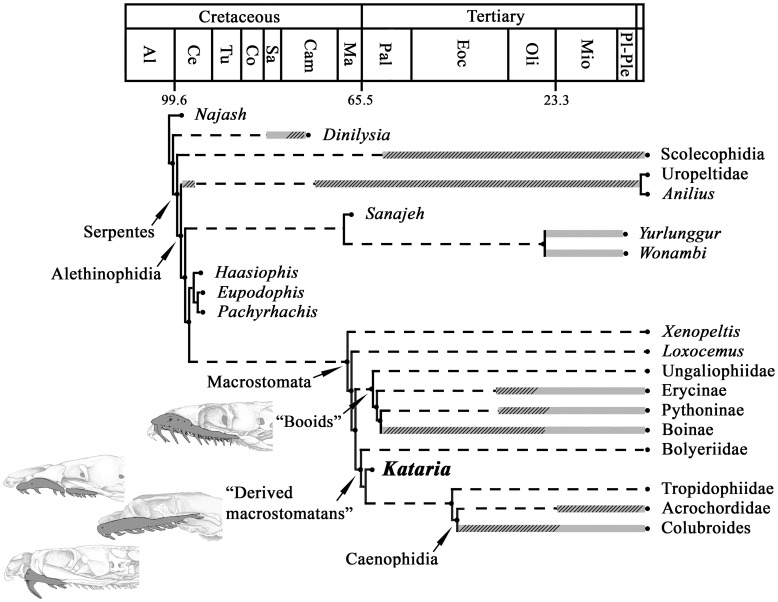
Phylogenetic relationships of *Kataria anisodonta.* Temporally calibrated cladogram of the most parsimonious tree obtained in this analysis. Thick gray lines indicate stratigraphic range of known taxa (dashed area indicates that these records are based on vertebral remains). Dashed lines represent ghost lineages implied by the stratigraphic distribution of fossils with respect to the phylogenetic relationships shown here (note the exceptionally abundant ghost lineages for Macrostomata). Ages of first appearance for taxa used in the calibrated phylogeny are given in electronic supplementary material. Al, Albian; Ce, Cenomanian; Tu, Turonian; Co, Coniacian; Sa, Santonian; Cam, Campanian; Ma, Maastrichtian; Pal, Paleocene; Eoc, Eocene; Oli, Oligocene; Mi, Miocene; Pl-Ple, Plio-Pleistocene.

The tree obtained from the phylogenetic analysis of parsimony (Fig. 4) placed *Kataria* deeply nested within derived macrostomatans, more precisely as the sister group of a clade composed by Tropidophiidae and Caenophidia. Our phylogenetic results also retrieve tropidophiids and caenophidians as sister taxa, in contrast to the basal alethinophidian position of the former in all recent molecular analyses [13], [14], [21], [30], [31], [32]. Despite the incompleteness of *Kataria* and of several other fossils added to the analysis, the obtained cladogram exhibits rather strong support values for several nodes (see electronic supplementary material).

As in more recent morphological analyses [2], [4], [5], [6], [7], [15], our results recovered the following two monophyletic sister-groups of advanced macrostomatans (i.e. excluding *Xenopeltis* and *Loxocemus*): 1) a clade composed by Ungaliophiidae+Erycinae+Boinae+Pythoninae, with a weak bootstrap support of 61% but moderate Bremer value of 5; and 2) a well-supported clade composed by bolyeriids, *Kataria*, tropidophiids, and caenophidian snakes, which received a strong bootstrap support of 82% but a low Bremer value of 3. This last group is supported by three unambiguous synapomorphies, all of which represent traits of the palatomaxillary arch: internal articulation of palatine with pterygoid short (70->0), ectopterygoid contact with the pterygoid is expanded significantly on the dorsal surface of the pterygoid body (76->1), and maxilla with posteromedial (ectopterygoid) expansions in the posterior region (156->1) (see Text S1 for a list of apomorphies for each clade).

**Figure 5:**
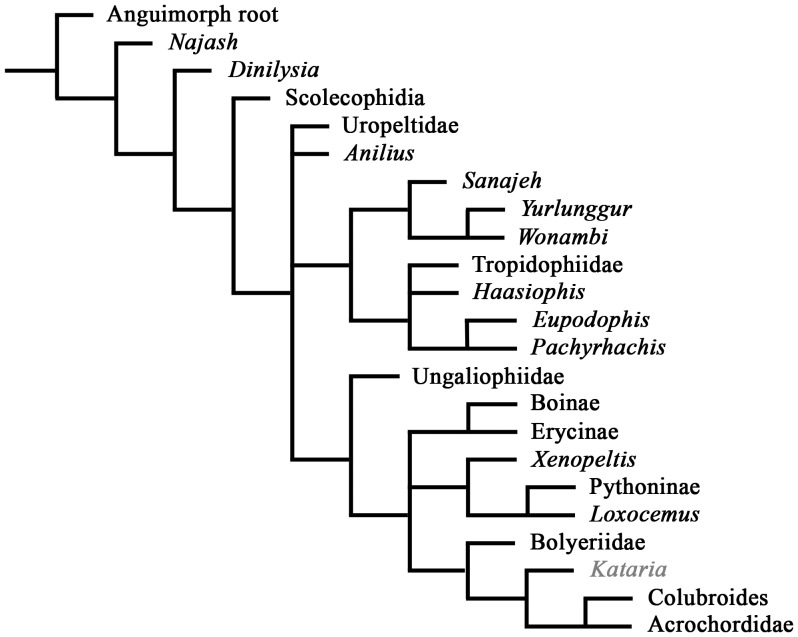
Strict consensus tree resulted from the constrained analysis.

Our additional constrained analysis conducted to test the effect of a molecular topology in the phylogenetic dataset resulted in two most parsimonious topologies. The strict consensus tree (Fig. 5) shows that all fossil taxa, except *Kataria*, were nested inside the enforced clade formed by the extant taxa *Anilius* and Tropidophiidae. *Kataria* retained its position as the sister-group of the clade formed by Acrochordidae and Colubroides, showing that enforcing a molecular tree as a backbone did not affect the position of *Kataria* as recovered in our previous unconstrained analysis.

## Discussion

### Morphology

With the exception of a few differences, the maxillary morphology of *Kataria* resembles that observed in many rear-fanged Colubroides, characterized by a tooth row composed of two toothed zones divided by a diastema. In a recent study on the development and evolution of snake fangs, Vonk et al. [16] proposed that the rear-fang condition found within colubroidean snakes is the product of a developmental decoupling of the dental maxillary lamina, recognizing the independent posterior dental lamina as responsible for the formation of the rear-fanged morphology characteristic of the endoglyptodont colubroideans (*sensu* [33]). The result of this developmental phenomenon is that the posterior region of the maxilla evolves independently, and exhibits conspicuous morphological differences with respect to the anterior maxillary region in postnatal individuals, especially in tooth morphology. The presence of a maxillary diastema and the distinct shape of the rear tooth in the maxilla of *Kataria* suggest that a similar developmental process occurred during the development of this bone in *Kataria*. Significantly, other derived macrostomatans show anatomical innovations in the maxilla beyond the plesiomorphic condition present in the rest of macrostomatans (Fig. 3). Within macrostomatan snakes, bolyeriids exhibit a maxilla divided in anterior and posterior parts by a transverse movable joint [29] while Colubroides display a tremendous variety of maxillary forms related to a venom-delivery system [11], [34], [35]. The peculiar maxillary shape of *Kataria* contrasts the conservative maxillary morphology of booids, indicating that the maxillary element might have played a relevant role in the early evolution of derived macrostomatans that was not necessarily associated with a venom delivery system.

Another feature of *Kataria* shared with tropidophiids, bolyeriids, and caenophidians, is the dorsal articulation between the ectopterygoid and pterygoid bones. The ectopterygoid bone has a crucial role in the highly derived feeding mechanisms of snakes [10], [11]. In macrostomatans with a lateromedial form of intraoral transport (booids), the lateral or laterodorsal immobile contact between these bones results in the functioning of each (left and right) palatomaxillary arch functions as a consolidated unit. In contrast, many colubroideans (including rear-fanged species) display a medial form of intraoral transport, with a freely movable joint that allows the ectopterygoid to swing rostrally or caudally in the horizontal plane during the translation of the palatopterygoid bar. These movements are permitted by the loose condition of the articular capsule, which even allows a slight degree of dorso-ventral movements [36], [37]. Thus, the palatopterygoid bar assumes the role of transporting (carrying) the prey during the intraoral transport of prey, which liberates the maxilla from an active role in prey intraoral transport [10]. Our analysis indicate that a medial form of intraoral transport appeared early in the history of derived macrostomatans, although myological studies in bolyeriids and tropidophiids indicate that the pterygoid musculature that produces the complex movements of the palatomaxillary arch necessary to act as medial transporters in caenophidians are not yet present in these groups [34].

It is widely assumed that the most important evolutionary innovation of macrostomatan snakes is the increase of gape size to swallow large items of food [10], [11], [12]. In this respect, our results suggest two different pathways in the evolution of the palatomaxillary arch of macrostomatan snakes. Booid snakes bear a lateromedial intraoral transport and no conspicuous modification in maxillary bone morphology. In contrast, small-bodied derived macrostomatans that freed their maxilla and pterygoid bones from a tight articulation with the ectopterygoid experienced drastic modifications in their maxillary morphology. The new function in active prey ingestion played by the palato-pterygoid arch of derived macrostomatans triggered important changes in the maxilla, including its tooth row morphology. *Kataria* represents the earliest documented record of such changes in maxillary tooth row morphology.

### Biogeographic Implications and Evolutionary Timing of Derived Macrostomatans

Recent discoveries of relevant fossil specimens in Mesozoic and Caenozoic strata have elucidated the central role of southern landmasses in the origin of snakes [2], [3], [4], [5], [6]. Moreover, the discovery of *Kataria* in South American bedrocks, together with the Neotropical distribution of extant tropidophiids and African (Seychelles archipelago) distribution of bolyeriids, suggest that the origin and early diversification of derived macrostomatans may also have taken place in Gondwanan terrains. These facts highlight the biogeographic importance of southern continents in the evolution of snakes, which was also pointed out by other lines of evidence such as molecular phylogenetics [32], [38].

Numerous snake materials assigned to different groups of macrostomatans have been found in Cretaceous and Paleocene deposits around the world [39], [40], [41], [42], [43]. However, it is worth noting that these records are represented by fragmentary remains, most of which composed by isolated vertebrae. The fragmentary condition of these specimens precludes rigorous phylogenetic analyses. Recent published work about the genus *Coniophis* represents an illustrative example of problems in the use of fragmentary snake material to determine phylogenetic relationships. *Coniophis* was previously known only by vertebral material scattered around the world and was historically classified as an “anilioid” alethinophidian. Using new material including skull elements, Longrich and colleagues [8] tested the phylogenetic relationships of *Coniophis* using a cladistic analysis and discovered that *Coniophis* constitutes a basal snake (i.e. a stem Serpentes), not an alethinophidian.

In light of these comments about the nature of the early fossil record of Macrostomata, *Kataria* emerges as the oldest calibration point for this entire clade of alethinophidian snakes tested through a resolved phylogenetic tree topology. Our temporally calibrated cladogram (Fig. 4) suggests that most cladogenetic events associated to the history of the clade Macrostomata, including the split between booids and derived macrostomatans, took place during Early Tertiary times at least. Also, the discovery of this new fossil snake indicates an unknown long history of the very distinctive families Bolyeriidae and Tropidophiidae, both representing important pieces of evidence to discern the evolutionary events of most derived forms of macrostomatan snakes.

## Supporting Information

Text S1 **Details of phylogenetic analyses and specimens examined.**
(DOC)

Dataset S1 **Nexus file containing the phylogenetic data matrix.**
(NEX)
