# Supplementary material for: A New Snake Skull from the Paleocene of Bolivia Sheds Light on the Evolution of Macrostomatans
Source: PLoS One. 2013 Mar 1;8(3):e57583. doi: 10.1371/journal.pone.0057583 (PMC3585969; doi:10.1371/journal.pone.0057583)
Supplement: Text S1 — Details of phylogenetic analyses and specimens examined. (DOC) [file pone.0057583.s001.doc]

Supplementary text for:

**“A new snake skull from the Paleocene of Bolivia sheds light on the evolution of macrostomatans”**

**Agustín Scanferla^1^, Hussam Zaher^2^, Fernando E. Novas^1^, Christian de Muizon^3^ and Ricardo Céspedes^4^**

*^1^ Laboratorio de Anatomía Comparada y Evolución de los Vertebrados. Museo Argentino de Ciencias Naturales “Bernardino Rivadavia”. Av. Angel Gallardo 470 (C1405DJR), Buenos Aires, Argentina.*

*² Museu de Zoologia, Universidade de São Paulo, Caixa Postal 42.494, São Paulo, Brasil.*

*³ UMR 8569 CNRS, Laboratoire de Paléontologie, 8, rue Buffon, F-75005 Paris, France.*

*^4^ Museo de Historia Natural “Alcide D´Orbigny”, Av. Potosí 1458 (4324), Cochabamba, Bolivia.*

Contents:

1.- Details of the phylogenetic analyses

1.1 List of characters employed 1

1.2 Data matrix 20

1.3 Heuristic tree search and support measures 24

2.-Ages of appearance used in calibrated phylogeny 28

3.- Specimens examined 29

4.- References 33

**1. Details of the phylogenetic analysis**

**1.1 List of characters employed**

The character-taxon matrix used in the phylogenetic analysis is mainly based on that proposed by [S1], with the addition of *Kataria* and 2 new characters. Thus, the purported analysis results in a data matrix of 156 characters scored across 23 taxa. All characters were treated as unordered, as in the original phylogenetic analysis. Capital letters “AZ”, “T”, “R”, “S”, “W” and ZS refer to characters used by Apesteguía and Zaher [S2], Tchernov et al. [S3], Rieppel, et al. [S4], Scanlon [S5], Wilson et al. [S6], and Zaher and Scanferla [S1] respectively, followed by the character number used by the authors. See [S1,S2,S5,S6] for additional information on the characters used herein.

**List of morphological characters**

**Dentition**

1. Tooth implantation on the dentary pleurodont (0), alethinophidian (in the sense of [S7]) (1) [AZ1, R1, W96, ZS1]. Although the maxillary teeth of *Kataria* show a condition that is clearly of the alethinophidian type, we prefer to code this terminal with a question mark in order to keep the definition of the character unchanged as it refers to teeth on the dentary (not preserved for *Kataria*). **(*Kataria* ?)**

2. Plicidentine (in the sense of [S8]) present (0), absent (1) [AZ2, R3, S172, W95, ZS2]. We modified the original definition of this character according to [6]. **(*Kataria* 1)**

3. Maxillary and dentary teeth relatively short conical and upright (0), robust and recurved (1), elongate needle-shaped, and distinctly recurved (2) [AZ3, R4, W94, ZS3]. **(*Kataria* 2)**

4. Premaxillary dentition present (0), absent (1) [AZ4, T1, S173, W1, ZS4]. **(*Kataria* ?)**

5. Alveoli and base of teeth not expanded transversely (0), wider transversely than anteroposteriorly (1) [AZ5, S178, ZS5]. **(*Kataria* 0)**

6. Pterygoid teeth absent (0), present (1) [T50, W69, ZS6]. **(*Kataria* 1)**

**Skull**

7. Premaxilla broadly articulated with the maxilla (0), or loosely contacting the maxilla (1) [S12, W4, ZS7]. *Kataria* is coded as 1 due to the rounded condition of the anterior tip of its maxilla, which suggests that the contact between premaxilla and maxilla was minimal or absent. **(*Kataria* 1)**

8. Transverse processes of premaxilla curved backwards (0), or extending straight laterally or anterolaterally (1) [AZ6, S11, W2, ZS8]. **(*Kataria* ?)**

9. Nasal process of premaxilla elongate, approaching or contacting frontals (0), short, divides nasals only at anterior margin or not at all (1) [S2, W3, ZS9]. **(*Kataria* ?)**

10. Dorsal (horizontal) lamina of nasal relatively broad anteriorly, leaving a narrow gap between its lateral margin and the vertical flange of the septomaxilla (0), or dorsal lamina of nasal distinctly tapering anteriorly, leaving a wide gap between its lateral margin and the vertical flange of the septomaxilla (1) [AZ7, T4, S25, W11, ZS10]. **(*Kataria* ?)**

11. Medial vertical flanges of nasals absent (0), or present (1). [ZS144]. **(*Kataria* ?)**

12. Vertical medial flanges of nasal do (0), or do not (1) articulate with the medial frontal pillars [AZ8, T5, W12, ZS11]. **(*Kataria* ?)**

13. Anterior margin of nasals restricted to posteromedial margins of nares (0), extends anteriorly toward tip of rostrum (1) [S24, W10, ZS12]. **(*Kataria* ?)**

14. Lateral flanges of nasal articulate with anterior margin of frontals (0), are separated from frontals (1) [S27, W13, ZS13]. **(*Kataria* ?)**

15. Posterolateral margin of nasal contacts posteromedian margin of prefrontal (0), elements in contact along most of their length (1), contact between elements with interfingering of nasal and prefrontal margins (2) nasals do not contact prefrontals (3) [S33, W14, ZS14]. **(*Kataria* ?)**

16. The posterior dorsal process of the lateral vertical flange of the septomaxilla is absent (0), short (1), or long (2) [AZ9, T6, S80, W84, ZS15]. **(*Kataria* ?)**

17. The septomaxilla does not (0), or does (1) articulate with the medial frontal pillars [AZ10, T7, S82, W85, ZS16]. In *Kataria*, the posterior tip of the septomaxilla is artificially displaced dorsally, giving the impression of a contact with the frontal. **(*Kataria* 0)**

18. Ventral portion of posterior edge of lateral flange of the septomaxilla is located at the level of the posterior edge or behind (0), or distinctly in front (1) of the opening of Jacobson’s organ [AZ11, T8, ZS17]. **(*Kataria* ?)**

19. Vomeronasal cupola fenestrated medially (0), or closed medially by a sutural contact of septomaxilla and vomer (1) [AZ12, T9, S86, W83, ZS18]. **(*Kataria* 1)**

20. The septomaxilla forms the entire lateral margin of the opening of Jacobson’s organ (0), or the vomer extends into the posterior part of the lateral margin, restricting the septomaxilla to the anterolateral part of the lateral margin of the opening of Jacobson’s organ (1) [AZ13, T10, S84, W79, ZS19]. **(*Kataria* 0)**

21. The vomeronasal nerve does not pierce the vomer (0), exits the vomer through a single large foramen (or an additional one or two smaller foramina) (1), or through a cluster of small foramina (2) [AZ14, T11, S85, W80, ZS20]. **(*Kataria* 1)**

22. Posterior ventral (horizontal) lamina of vomer long, parallel edged (0), or short, tapering to a pointed tip (1) [AZ15, T12, S88, W81, ZS21]. **(*Kataria* 0)**

23. Posterior dorsal (vertical) lamina of vomer well developed (0), or reduced or absent (1) [AZ16, T13, S89, W82, ZS22]. **(*Kataria* 0)**

24. Prefrontal articulates with the frontal laterally (0), or anterolaterally (1) [S41, W22, ZS23]. **(*Kataria* 1)**

25. Outer orbital (lateral) margin of the prefrontal is slanting anteroventrally (0), or is positioned vertically (1) [AZ17, S39, ZS24]. **(*Kataria* 1)**

26. The lacrimal foramen on the prefrontal is not completely enclosed (0), or is completely enclosed by the prefrontal (1) [AZ18, S45, W20, ZS25]. **(*Kataria* 0)**

27. The lateral foot process of the prefrontal is absent (0), contacts the maxilla only (1), or the maxilla and the palatine (2), or the palatine only (3) [AZ19, S36, W18, ZS26]. The definition of this character was modified according to [6]. **(*Kataria* 1)**

28. The medial foot process of the prefrontal (in the sense of [S9: fig. 18] is absent (0), present, low (1), or present, high (2) [AZ20, T17, W19, ZS27]. **(*Kataria* 1)**

29. Anterior / lateral flange of prefrontal, covering completely the nasal gland and roofing the aditus conchae, is absent (0), or well developed (1) [AZ21, T19, W15, ZS28]. **(*Kataria* 0)**

30. Lateral foot process of the prefrontal articulates with the lateral edge of the maxilla via a thin anteroposteriorly directed lamina (0), or articulates with the maxilla via a large contact that runs from the lateral to the medial dorsal surface of the maxilla (1). [ZS146]. **(*Kataria* 1)**

31. The ventral margin of the lateral surface of the prefrontal articulates along its entire length with the dorsal surface of the maxilla (0), or retains only a posterior contact (1) (i.e., at the anteroventral corner of the orbit) [AZ22, T22, S34, W17, ZS29]. In *Kataria*, the anterior tip of the lateral flange is not preserved. However, the dorsomedial surface of the maxilla still retains a surface of contact that would have received the ventral edge of the lateral flange. **(*Kataria* 1)**

32. The dorsal lamina of the prefrontal contacts or forms an overlapping contact with the nasal posteromedially (0), or remains separate from the nasal (1) [AZ23, T23, S33, ZS30]. *Yurlunggur* is coded with a question mark since the nasals and prefrontals seem to retain only incompletely preserved edges. **(*Kataria* ?)**

33. Medial frontal pillars absent (0), present and projected ventrally (1) [AZ24, T24, R54, S56, W21, ZS31]. **(*Kataria* 1)**

34. Frontal subolfactory process (lateral frontal pillar) absent or present as a simple horizontal lamina (0), or present and closing the tractus olfactorius medially (1). [ZS150]. **(*Kataria* 1)**

35. Preorbital process dorsally exposed (0), or overlapped by the prefrontal (1). [ZS145]. **(*Kataria* 1)**

36. Transverse horizontal shelf of the frontal (in the sense of [S9]) developed and broadly overlapped by nasals (0), or poorly developed and never broadly overlapped by nasals (1), absent (2) [AZ25, T20, S53, ZS32]. **(*Kataria* 1)**

37. Lacrimal present (0), absent (1) [W16, ZS33]. **(*Kataria* 1)**

38. Postfrontal present (0), or absent (1) [AZ26, T25, ZS34]. **(*Kataria* 1)**

39. Postorbital present (0), or absent (1) [AZ27, T26, S47, ZS35]. **(*Kataria* 0)**

40. Ventral tip of postorbital remains separated by a wide gap from ectopterygoid (0), or it touches (in dried skulls), or closely approaches the ectopterygoid, forming an almost complete posterior margin of the orbit (1) [AZ28, T27, S51, W25, ZS36]. **(*Kataria* 0)**

41. The dorsal head of the postorbital fuses or articulates with the posterodorsal surface of the postfrontal (0), articulates with the parietal (1) [AZ29, T28, S48, W24, ZS37]. **(*Kataria* 1)**

42. Parietal without lateral wings meeting postorbital bones (0), or with lateral wings meeting postorbital bones (1) [AZ30, R14, S50, W27, ZS38]. **(*Kataria* 1)**

43. Distinct lateral ridge of parietal extending posteriorly from anterior lateral wing up to prootic is absent (0), or present (1) [AZ31, R15, S67, W29, ZS39]. **(*Kataria* 0)**

44. Fronto-parietal suture relatively straight (0), or distinct supraorbital process of parietal extends along at least 50% of the length of dorsal margin of orbit (1) (i.e., fronto-parietal suture U-shaped) [AZ33, T32, S54, W23, ZS40]. **(*Kataria* 0)**

45. The parietal margin of the optic foramen is straight (0) (i.e., the parietal is not notched by the optic foramen), or concave (1) (i.e., the parietal is notched by the optic foramen) [AZ34, T33, S61, W30, ZS41]. **(*Kataria* 1)**

46. Lateral margins of the braincase open anterior to prootic (0), descending lateral processes of parietal enclose braincase (1) [W28, ZS42]. **(*Kataria* 1)**

47. Supratemporal processes of parietal distinctly developed (0) (i.e., parietal expanded at level of jaw suspension), or not distinctly developed (1) [AZ35, T34, S66, W33, ZS43]. **(*Kataria* 1)**

48. Parietal does not (0), or does (1) enter the anterior aspect of the base of the basipterygoid process along its entire width [AZ36, T36, S68, ZS44]. **(*Kataria* ?)**

49. Contact between parietal and supraoccipital is V-shaped with the apex pointing anteriorly (i.e., the parietal is embayed posteriorly) (0), or is essentially a straight transverse line (1), or is V-shaped with the apex pointing posteriorly (i.e., posteriorly pointed) (2) [AZ37, T37, S65, ZS45]. **(*Kataria* ?)**

50. Ascending process of the maxilla present (0), absent (1) [AZ38, T29, R5, S14, W6, ZS46]. **(*Kataria* 1)**

51. Small horizontal shelf on medial surface of anterior end of maxilla present (0), absent (1) [AZ40, R7, S15, W5, ZS47]. **(*Kataria* 1)**

52. Posterior end of maxilla does not project beyond posterior margin of orbit (0), projects moderately beyond posterior margin of orbit (1), projects distinctly beyond posterior margin of orbit, with broad flat surface (2) [AZ41, R8, S23, W9, ZS48]. **(*Kataria* 2)**

53. Medial (palatine) process of maxilla located in front of the orbit (0), or located below the orbit (1) [AZ42, R9, S18, W7, ZS49]. **(*Kataria* 0)**

54. Medial (palatine) process of maxilla pierced (0), or not pierced (1) [AZ43, T30, S21, W8, ZS50]. **(*Kataria* 1)**

55. Anterior end of supratemporal (facet) located behind or above posterior border of trigeminal foramen (0), or in front of posterior border of trigeminal foramen (1) (AZ44, T40, R30, S72, W36, ZS51]. **(*Kataria* ?)**

56. Supratemporal facet on opisthotic-exoccipital flat (0), or sculptured and delineated with projecting posterior rim that overhangs exoccipital (1) [AZ45, R31, W38, ZS52]. **(*Kataria* ?)**

57. Free-ending posterior process of supratemporal absent (0), present (1) [AZ46, T39, R32, S70, W35, ZS53]. **(*Kataria* ?)**

58. Supratemporal present (0) or absent (1) [AZ47, T38, S69, W34, ZS54]. **(*Kataria* ?)**

59. Anterior dentigerous process of palatine absent (0), present (1) [AZ48, T41, R10, S94, W72, ZS55]. **(*Kataria* 1)**

60. Maxillary process of the palatine represents the main element that bridges the contact between maxilla and palatine in a ventral view (0), or is covered ventrally by an expanded palatine process of the maxilla. [ZS152]. **(*Kataria* 1)**

61. Medial (choanal) process of palatine forms an extensive concave surface dorsal to the ductus nasopharingeus (0), narrows abruptly to form a curved finger-like process (1), or forms a short horizontal lamina that does not reach the vomer (2) [AZ49, T48, R11, S91-92, W75, ZS56] **(*Kataria* 0)**

62. Choanal process of palatine without expanded anterior flange articulating with vomer (0), with anterior flange (1) [S93, W76, ZS57]. **(*Kataria* 0)**

63. Pterygoid contacts palatine in more or less complex pattern with clasping projections (0), in tongue-in-groove joint (1), or palatine-pterygoid contact reduced to flap-overlap (2) [AZ50, R12, S99, W77, ZS58]. **(*Kataria* ?)**

64. Palatine does (0), or does not (1) contact the ectopterygoid [AZ51, T42, W78, ZS59]. **(*Kataria* 1)**

65. The dentigerous process of the palatine meets the vomer and/or septomaxilla posterolateral to the opening of Jacobson’s organ (0), or fails to do so (1) [AZ52, T43, ZS60]. **(*Kataria* 1)**

66. Lateral (maxillary) process of palatine is situated anterior to the posterior end of palatine (0), or at the posterior end of the palatine (1) [AZ53, T44, S97, W74, ZS61]. **(*Kataria* 0)**

67. Lateral (maxillary) process of palatine in well-defined articulation (0), or only loosely overlapping the medial (palatine) process of maxilla, or no articulation between palatine and maxilla (1) [AZ54, T45, S96, ZS62]. **(*Kataria* 1)**

68. Maxillary branch of trigeminal nerve pierces the lateral (maxillary) process of the palatine (0), or it passes dorsally between the palatine and the prefrontal (1) [AZ55, T46, S98, W73, ZS63]. **(*Kataria* 0)**

69. The vomerine (choanal) process of the palatine articulates broadly with the posterior end of the vomer, being continuous with the latter (0), meets the vomer in a well-defined articular facet (1), touches or abuts the vomer without articulation or remains separated from the vomer (2) [AZ56, T47, S90, ZS64]. **(*Kataria* ?)**

70. Internal articulation of palatine with pterygoid short (0), or long (1) [AZ57, T49, S99, ZS65]. **(*Kataria* ?)**

71. Pterygoid tooth row lies ahead of the basipterygoid joint (0), tooth row reaches or passes shortly the level of the basipterygoid joint posteriorly (1), extends greatly along the medial margin of the quadrate ramus of the pterygoid bone (2) [AZ58, T50, R13, ZS66]. The original definition [AZ58, T50, R13] is slightly modified here in order to allow appropriate coding of colubroidean terminals (in the sense of [S10]). **(*Kataria* ?)**

72. Quadrate ramus of pterygoid robust, rounded or triangular in cross-section, but without groove (0), or blade-like and with distinct longitudinal groove for the insertion of the muscle protractor pterygoidei (1) [AZ59, T51, S103-105, ZS67]. **(*Kataria* ?)**

73. Transverse (lateral) process of the pterygoid forms a distinct, well-defined lateral projection (0), is a gently curved lateral expansion of the pterygoid or is absent (1) [AZ60, T52, 101, S101, W70, ZS68]. **(*Kataria* ?)**

74. The lateral edge of the ectopterygoid is straight (0), or clearly angulated at the level of its contact with the maxilla (1) [AZ61, T53, S113, ZS69]. We corrected [S1] coding of Bolyeriidae from state 0 to state 1. **(*Kataria* 1)**

75. Anterior end of ectopterygoid restricted to posteromedial edge of maxilla (0), or invades the dorsal surface of the maxilla (approaching the posteroventral corner of the orbit) (1) [AZ62, T54, S111, W71, ZS70]. **(*Kataria* 1)**

76. The ectopterygoid contact with the pterygoid is restricted to the transverse (lateral) process of the pterygoid (0), contact with the pterygoid is expanded significantly on the dorsal surface of the pterygoid body (1). [ZS151]. **(*Kataria* 1)**

77. Medial finger-like process of the ectopterygoid articulating with the medial surface of the maxilla present (0), or absent (1). [ZS147]. **(*Kataria* 1)**

78. Pterygoid attached to basicranium by strong ligaments at palatobasal articulation (0), or pterygoid free from the basicranium in dried skulls (i.e. supported by cid-muscles) (1) [AZ63, T83, ZS71]. **(*Kataria* ?)**

79. Quadrate slender (0), or broad, with a rectangular shape (1) [AZ64, ZS72]. **(*Kataria* ?)**

80. Quadrate slanted clearly anteriorly, posterior tip of pterygoid dislocated anteriorly from mandibular condyle of quadrate (0), positioned slight anteriorly or vertically (cephalic condyle positioned behind or at the same level of mandibular condyle) (1), slanted posteriorly (cephalic condyle positioned in front of mandibular condyle) (2) [AZ65, T55, S78, W41, ZS73]. **(*Kataria* ?)**

81. Cephalic condyle of quadrate elaborated into posteriorly projecting suprastapedial process (0), or suprastapedial process absent or vestigial (in the adult) (1) [AZ66, T56, S74, W40, ZS74]. **(*Kataria* ?)**

82. Stapedial footplate broad and massive (0), narrow and thin (1) [W53, ZS75]. **(*Kataria* ?)**

83. Stylohyal not fused to the quadrate (0), fuses to the posterior tip of the suprastapedial process of quadrate (1), fuses to the ventral aspect of a reduced (embryonic) suprastapedial process (2), or the stylohyal fuses directly to the shaft of the quadrate (3) [AZ67, T59, S73, W42, ZS76]. **(*Kataria* ?)**

84. Stapedial shaft straight (0), or angulated (1) [AZ68, T60, S144, W51, ZS77]. **(*Kataria* ?)**

85. Stapedial shaft slender and longer than diameter of stapedial foot-plate (0), or thick, and equal to, or shorter than diameter of stapedial foot-plate (1) [AZ69, T61, S145, W52, ZS78]. **(*Kataria* ?)**

86. Paroccipital process of otooccipital well developed and laterally projected (0), reduced to a short projection or absent (1) [AZ70, T73, R33, S137, W50, ZS79]. **(*Kataria* ?)**

87. Juxtastapedial recess defined by crista circumfenestralis absent (0), present but open posteriorly (1), or present and closed posteriorly (2) [AZ71, T74, S135, R34, W45, ZS80]. **(*Kataria* ?)**

88. Crista circumfenestralis exposes most of stapedial footplate (0), converges upon stapedial footplate (1) [AZ72, R35, ZS81]. **(*Kataria* ?)**

89. Crista interfenestralis does not form individualized component in ventral rim of crista circumfenestralis (0), does form individualized component in ventral rim of crista circumfenestralis (1) [AZ73, R36, W48, ZS82]. **(*Kataria* ?)**

90. Jugular foramen exposed in lateral view by crista tuberalis (0), concealed in lateral view by crista tuberalis (1) [AZ74, R37, W47, ZS83]. **(*Kataria* ?)**

91. Otooccipitals do not contact each other dorsally (0), contact each other dorsally (1) [AZ75, S141, W49, ZS84]. **(*Kataria* ?)**

92. Otooccipital posterolateral processes short and narrow, do not extend toward posterior margin of occipital condyle (0), wider than condyle and long, combine with crista tuberalis to extend to approximate posterior margin of occipital condyle (1) [W57, ZS85]. **(*Kataria* ?)**

93. Supraoccipital with narrow (i.e., less than parietal) (0), or broad (i.e., as long as or more than parietal) contact to prootic (1) [AZ76, T62, S140, ZS86]. **(*Kataria* ?)**

94. Prootic does not exclude parietal from trigeminal foramen (0), does exclude parietal from trigeminal foramen (1) [AZ77, T70, R27, S132, W31, ZS87]. **(*Kataria* ?)**

95. Laterosphenoid absent (0), present (1) [AZ78, T65, R28, S130, W43, ZS88]. **(*Kataria* ?)**

96. Posteriorly undercut prootic ledge underlap posterior trigeminal foramen absent (0), present (1) [AZ79, R29, ZS89] **(*Kataria* ?)**

97. Prootic exposed in dorsal view medial to the supratemporal or to supratemporal process of parietal (where supratemporal is lacking) (0), or fully concealed by the supratemporal or parietal in dorsal view (1) [AZ80, T66, S136, W46, ZS90]. **(*Kataria* ?)**

98. Exit foramen for the hyomandibular branch of the facial nerve is located outside (0), or inside (1) the opening for the mandibular branch of the trigeminal nerve [AZ81, T67, S133, W44, ZS91]. **(*Kataria* ?)**

99. Vidian canal does not open intracranially (0), open intracranially (1) [AZ82, R17, S124, W66, ZS92]. **(*Kataria* ?)**

100. Anterior opening of Vidian canal single (0), or divided (1) [AZ83, R18, ZS93]. **(*Kataria* ?)**

101. Sella turcica bordered posteriorly by well-developed dorsum sellae (0), dorsum sellae low (1), dorsum sellae not developed, sella turcica with shallow posterior margin (2) [AZ84, R20, S128, W67, ZS94]. **(*Kataria* ?)**

102. "Lateral wings of the basisphenoid" absent (0), present (1) [AZ85, T79, R21, S119, W65, ZS95]. **(*Kataria* ?)**

103. Ventral surface of the basisphenoid smooth (0), with weakly developed sagittal crest from which protractor pterygoidei originates (1), with strongly projecting sagittal crest (2) [AZ86, T77, S120, R22, W54-68, ZS96]. **(*Kataria* ?)**

104. Basioccipital contributes to ventral margin of foramen magnum (0), basioccipital excluded by medial contact of otooccipitals (1) [S142, W58, ZS97]. **(*Kataria* ?)**

105. Basisphenoid-basioccipital suture smooth (0), transversely crested (1) [AZ87, R23, W55, ZS98]. **(*Kataria* ?)**

106. Basipterygoid (=basitrabecular) processes present (0), absent (1) [AZ88, T82, R24, S117, W63-64, ZS99]. **(*Kataria* ?)**

107. Crista trabecularis short and or indistinct (0), elongate and distinct in lateral view of the basisphenoidal rostrum (1) [AZ89, R25, W62, ZS100]. **(*Kataria* ?)**

108. Cultriform process of parabasisphenoid does not extend anteriorly to approach posterior margin of choanae (0), approaches posterior margin of vomera (1) [W59, ZS101]. **(*Kataria* 1)**

109. Posterolateral corners of the basisphenoid strongly ventrolaterally projected (0), or not projected (1). [ZS148]. **(*Kataria* ?)**

110. Para-basisphenoidal rostrum behind optic foramen narrow (0), broad (1) (AZ90, T80, R26, S116, W60, ZS102]. **(*Kataria* 0)**

111. Parabasisphenoid rostroventral surface flat or broadly convex (0), concave (1) [S116, W61, ZS103]. **(*Kataria* 1)**

112. Basioccipital expanded laterally to form the floor of the recessus scalae tympani (0) or excluded from the floor of the recessus scalae tympani by the otooccipital (1). [ZS149]. **(*Kataria* ?)**

113. Basioccipital meets para-basisphenoid in a suture located at the level of the fenestra ovalis (0); or located at level or just behind the trigeminal foramen (foramina) (1) [AZ91, T78, S121, W56, ZS104]. **(*Kataria* ?)**

114. Parasphenoid rostrum without (0), or with broad based (1), or narrow based (2) interchoanal process [AZ92, T80, S115, W60, ZS105]. **(*Kataria* 2)**

**Mandible**

115. Anteromedial margin of dentaries possess symphyseal articular facet (0), dentaries lack symphyseal articular facet (1) [W86, ZS106]. **(*Kataria* ?)**

116. Posterior dentigerous process of dentary absent (0), present and short (1), present and long (2) [AZ93, T87, R38, S150, W88, ZS107]. **(*Kataria* ?)**

117. Medial margin of adductor fossa relatively low and smoothly rounded (0), forms a distinct dorsally projecting crest (1) [AZ94, T88, R39, S166, W93, ZS108]. **(*Kataria* ?)**

118. Mental foramina on lateral surface of the dentary two or more (0), one (1) [AZ95, S148, W87, ZS109]. **(*Kataria* ?)**

119. Coronoid process of coronoid bone high, tappering distally (0), high, with a rectangular shape (1), low, not exceeding significantly the coronoid process of the compound bone (2) [AZ96, T86, S164, ZS110]. **(*Kataria* ?)**

120. Coronoid bone present (0) absent (1) [AZ97, T84, S160, W89, ZS111]. **(*Kataria* ?)**

121. Coronoid bone contributes to the anterior margin of the adductor fossa (0), does not form (reach) the anterior margin of the adductor fossa (1). [ZS153]. **(*Kataria* ?)**

122. Coronoid bone sits mostly on the dorsal and dorsomedial surfaces of the compound bone, being exposed in both lateral and medial views of the mandible (0), or is applied to the medial surface of the compound bone (1). [ZS154]. **(*Kataria* ?)**

123. Posteroventral process of the coronoid present (0), absent (1) [AZ98, T85, S161, W91, ZS112]. **(*Kataria* ?)**

124. Coronoid process on lower jaw formed by coronoid bone only (0), or by coronoid and compound bone (1), or by compound bone only (2) (i.e., coronoid absent) [AZ99, T86, S160-164, W90, ZS113]. **(*Kataria* ?)**

125. Discrete surangular and articular postdentary elements (0), fusion of surangular and articular into compound bone (1) [W92, ZS114]. **(*Kataria* ?)**

**Vertebrae**

126. Chevrons present (0), absent (1) [AZ100, R41, S204-205-206, ZS115]. **(*Kataria* ?)**

127. Hemapophyses absent (0), present (1) [AZ101, R42, S204-205-206, W110, ZS116]. **(*Kataria* ?)**

128. Hypapophyses restricted to anterior most precloacal vertebrae (0), present throughout precloacal skeleton (1) [S201, W107, ZS117]. **(*Kataria* ?)**

129. Para-diapophysis confluent (0), separated into dorsal and ventral facet (1) [AZ102, R43, ZS118]. **(*Kataria* ?)**

130. Prezygapophyseal accessory processes absent (0), present (1) [AZ103, R44, S200, W98, ZS119]. **(*Kataria* ?)**

131. Subcentral paralymphatic fossae on posterior precloacal vertebrae absent (0), present (1) [W108, ZS120]. **(*Kataria* ?)**

132. Subcentral foramina absent (0), present and consistently small (1), present, of variable size (2) [AZ104, R45, S199, W104, ZS121]. **(*Kataria* ?)**

133. Well-developed, consistently distributed paracotylar foramina absent (0), present (1) [W97, ZS122]. **(*Kataria* ?)**

134. Ventral margin of centra smooth (0), median prominence extending from cotyle to condyle on ventral surface (1) [S202, W105, ZS123]. **(*Kataria* ?)**

135. Second (axis) intercentrum not fused to anterior region of axis centrum, suturally connected at most (0), fused to anterior region of axis centrum (1) [AZ105, S189, ZS124]. **(*Kataria* ?)**

136. Neural spine height, a well-developed process (0), low ridge or absent (1) [AZ106, S190, W101, ZS125]. **(*Kataria* ?)**

137. Posterior margin of neural arch shallowly concave in dorsal view (0), with deep V-shaped embayment in dorsal view exposing much of centrum in front of condyle (1) [AZ107, S191, W102, ZS126]. **(*Kataria* ?)**

138. Cotyle shape of precloacal vertebrae strongly oval (0), approximately circular (1) [AZ108, S193, W100, ZS127]. **(*Kataria* ?)**

139. Parazygantral foramen absent (0), present (1) [AZ109, S198, W103, ZS128]. **(*Kataria* ?)**

140. Lymphapophyses absent (0), present (1) [AZ110, S203, W109, ZS129]. **(*Kataria* ?)**

141. Lymphapophyses, three or fewer (0), three lymphapophyses and one forked rib (1), more than three lymphapophyses and one forked rib (2) [AZ111, S203, W109, ZS130]. **(*Kataria* ?)**

142. Sacral vertebrae present (0), absent (1) [AZ112, ZS131]. **(*Kataria* ?)**

143. Position of synapophyses in relation to lateral edge of prezygapophyses at the same level or slightly more projected laterally (0), clearly medial to the edge of the prezygapophyses (1) [AZ113, W99, ZS132]. **(*Kataria* ?)**

144. Pachyostotic vertebrae absent (0), present (1) [AZ114, S208, ZS133]. **(*Kataria* ?)**

145. Precloacal vertebrae number less than 100 (0), more than 100 (1) [W106, ZS134]. **(*Kataria* ?)**

146. Number of caudal vertebrae. 0: more than 30. 1: fewer than 30 [S187, W111, ZS135]. **(*Kataria* ?)**

147. Tuber costae absent from ribs (0), tuber costae present (1) [S207, W112, ZS136]. **(*Kataria* ?)**

**Hindlimbs**

148. Pectoral girdle and forelimbs present (0), absent (1) [W113, ZS137]. **(*Kataria* ?)**

149. Tibia, fibula, and hind foot present (0), absent (1) [S212, W114, ZS138]. **(*Kataria* ?)**

150. Trocanter externus present (0), absent (1) [AZ115, S212, W115, ZS139]. **(*Kataria* ?)**

151. Pelvis external to sacral-cloacal ribs (0), internal to sacral-cloacal ribs (1) [AZ116, S211, W116, ZS140]. **(*Kataria* ?)**

152. Ilium and pubis length, ilium longer than pubis (0), ilium and pubis of same size (1), pubis much longer than ilium (2) [AZ117, ZS141]. **(*Kataria* ?)**

153. Pelvic elements with strongly sutured contact (0), with weak (cartilaginous) contact (1), fused together (2) [AZ118, ZS142]. **(*Kataria* ?)**

154. Pelvic elements present (0), absent (1) [AZ119, W116, ZS143]. **(*Kataria* ?)**

**Additional characters**

155. Maxillary dentition composed by several unmodified teeth that decrease uniformly in size posteriorly (0), or slightly to greatly enlarged teeth are present in the posterior tip of maxillary tooth row (1). (Discussed by [S11,S12,S13]). **(*Kataria* 1)**

156. Maxilla without distinct posteromedial (ectopterygoid) expansion or flange (0), or this bone bears a weak but distinct posteromedial (ectopterygoid) expansion or flange (1), or the maxilla with large posteromedial (ectopterygoid) expansion or flange (2). [S22]. **(*Kataria* 1)**

**1.2 Data matrix**

Anguimorph_Root

00001(01)00010-00(03)0?00000100000000(01)0(01)?000000000-00010000-000000 0000-0000000010000010100000--100?0000-0000000-000000000000000000000000 0(01)00000000-000000000000000

Najash

0???1?????????????????????????????????????0??1000?????00?0?????????????0???????100???010?00?000000???00?000??11?0?0??0???????11010?1?101011100001?1?000110??

Dinilysia

111?111??10-100??????0?01021010000001001011001001001000000000?01-0000 000000000010001101010100000001(01)100000?1011000110?0000001??011?(01)01?0010???101?1????????0

Scolecophidia

01011011101-1010??000??00???0?0000?211(01)??000-11?10?(01)1?000(01)00(01)0 20---1(01)0-0100(01)10000111112100100000?0?0200001011111(01)0100000(01)1 00110001120(01)(01)10(01)01(12)11011111(01)111000

Anilius

11101110001010020000100101110100111111101001010?100100000011001100011110000000010111101000100110001021000111101101110120110111101111010101011110111111111000

Uropeltidae

11111010(01)01010(01)100(01)0100100110100111111001001010?100100000(01)(01)10011000011100000000101111(01)10001000100010210(01)0(01)(01)1111101110120110111101111010101011110111111111000

Sanajeh

111???????????????????????2?????????1?????00?10?00020?0010??0??1???1??????1??????????01?1111100?10???12?10??1?011?12?1??????1??010???1?0111???001?1????????0

Wonambi

112?111??????0?????????0????????1??1?????110110010120011?01?0111?011111?0?10?????????01011110000?0011121101?11111?1201?0????1?0010?111?01111??001?1???????00

Yurlunggur

112111101?1?103??0?01000001?0?0?100110010011010??0020001?01101?100111?0001100?010????01011?1?000?0011121101111111?12?0??????1??010111100111???001(01)1???????00

Pachyrhachis

112??11?1???10?????????00???0?0?????110111?0?11??012???0101???11??????101110??111?20?1??????????1??????????1?0????120110??001??0101111?001?121111?1101111000

Haasiophis

1121111?1???10???????0?00???0?0?1???110110?1?11?00121?001011??11?11??11?1110??011????1????0???111?????1?0??1?0????12010011001110101111?10??121?1101101111000

Eupodophis

112?011?1???10????????????1?????1????1011????1???0101???101?1?11????20101??0??1?1????1????????1???????1????1??????120110??001100001111?00??0-111111 1011?1000

Xenopeltis

112001101010102200101001103101001111111??001011101020100101110110011111111101001000111101010001010101110011110011212012011111110111101110101211010111----101

Loxocemus

112001101110102100101001102101001111100010011111010201101011101100111111111010011121011010100(01)101110111011111001121201201111111011110111010121101011111?2000

Erycinae

11210111111010(01)1011011011012(01)11011111(01)0(01)1100111(01)(12)111101010(01)11011111121111(01)1010011120012011100(01)1011(01)?11(12)0(01)0111 (01)0110121120111111101111010011012110101111122000

Ungaliophiidae

112101101110100201101101(01)0120110111111001100111?011(02)10001011101111112(01)111010100111200120101000101110110001111001121211-1??-211101111010011012110101111122000

Boinae

1121011(01)111010(01)2011011011(01)1211101111110111(01)01111211210111011(12)01111112011111010011120012111(01)00(01)11110111201011100112121120111111101111(01)10011012110101111122000

Pythoninae

112(01)011(01)111010020010110111121110111110011100111?01121011101120111(01)102111111010011120012111100(01)101(01)00112(01)1011100112121120111111101111010011012110101111122000

Tropidophiidae

11210111111011320010100110110111111111011100111?011010101011001110112011101110011120012011101110111011100111100112121120111211111111110011011110101111122(01)01

Bolyeriidae

11210110111011320010100110110110111111001100111?01120110101110111011201111111001112001201110001011??1100011110011212112011111110111111001101211010111----101

Acrochordidae

11210111101111310011211111200111111111011110-11?01121110101100111011 201111111102112001???01011101110111001111001101200?1??-21111111111(01) 01101211010(01)11----101

Colubroides

1121011(01)1(01)1(01)113110112111(01)0(02)(01)(01)111111111(01)01100111?011(012)(01)11010110011101020111(01)111102113001211110111011(01)(01)1(01)00011110(01)1101211-1---211111110110(01)110121101011?----1(01)2

Kataria

?12?011?????????0?1010011011011?111111001100111??11201????1100?11010?????1111?????????????????????????????11?01??2????????????????????????????????????????11

**1.3 Heuristic tree search and support measures**

The character-taxon matrix was assembled in Mesquite v.2.6 [S14]. We analysed our dataset using TNT [S15] with a heuristic search of 1000 replicates of Wagner trees followed by tree bisection-reconnection (TBR) branch swapping. All characters were equally weighted. Zero length branches were collapsed if they lack support under any of the most parsimonious reconstructions. Also, two alternative support measures (Bremer support and bootstrap resampling) were used to evaluate the robustness of the nodes of the obtained most parsimonious trees. The parsimonious analysis resulted in a one most parsimonious tree of 341 steps (CI = 0,54 - RI = 0,7).

**1.3.1 Obtained tree**


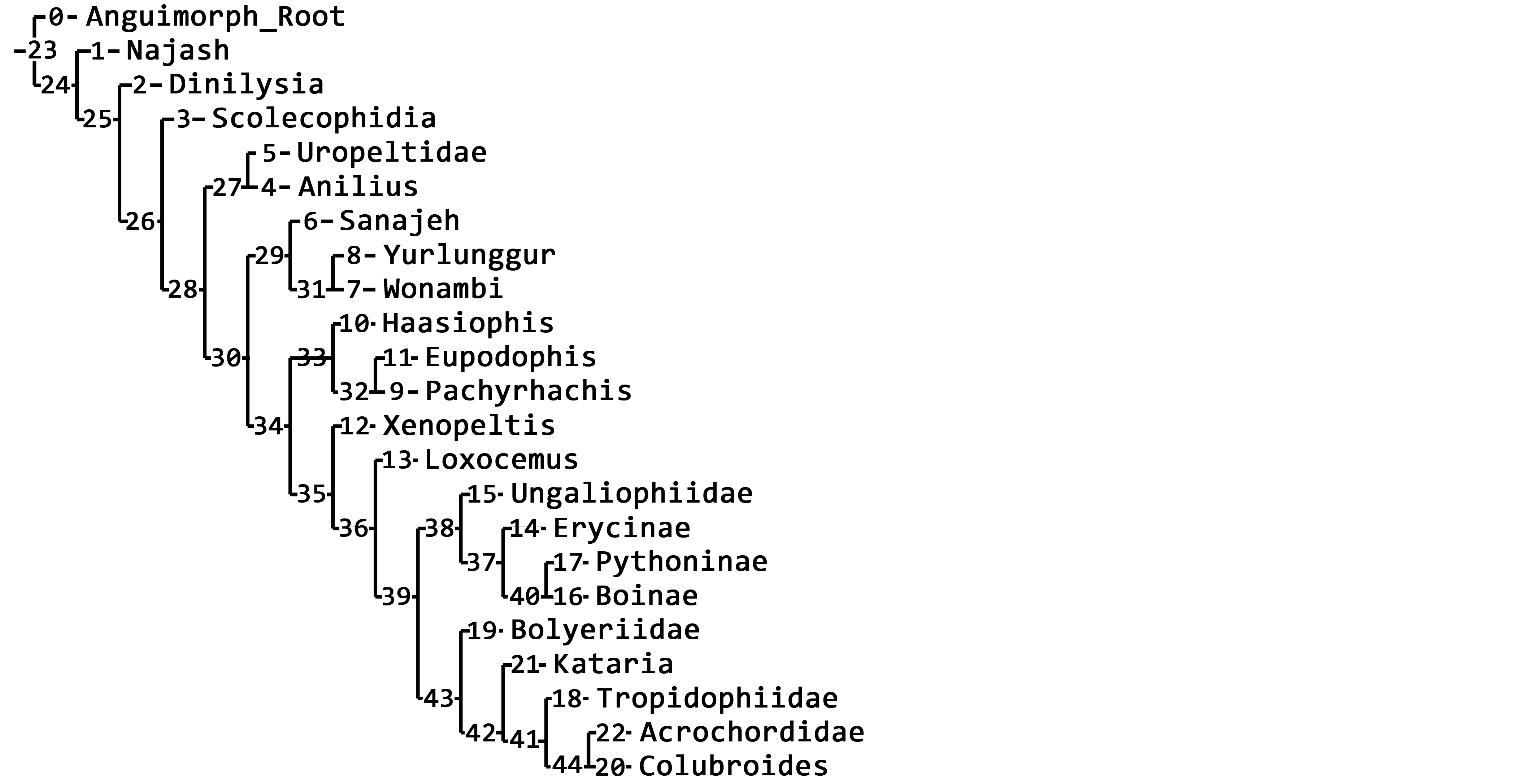


**1.3.2 List of synapomorphies for the labelled nodes:**

**Node 25**: 91 (1), 115 (1), 130 (1), 143 (1). **Node 26**: 10 (0), 11 (1), 38 (1), 68 (1), 83 (1), 106 (1), 109 (1), 112 (1), 122 (1). **Node 27**: 24 (1). **Node 28**: 16 (2), 21 (1), 33 (1), 59 (1), 60 (1), 102 (1), 107 (1), 118 (1). **Node 29**: 90 (1), 92 (1), 105 (1), 106 (0), 136 (0), 137 (1), 139 (1), 143 (0). **Node 30**: 52 (2), 57 (1), 67 (1), 74 (1), 75 (1), 97 (1), 113 (1). **Node 31**: 43 (1), 56 (1). **Node 32**: 79 (1), 136 (0). **Node 33**: 51 (1), 53 (1), 144 (1), 149 (0). **Node 34**: 47 (1), 61 (1), 73 (1), 86 (1), 110 (0). **Node 35**: 24 (1), 25 (1), 50 (1), 72 (1), 123 (1). **Node 36**: 10 (1), 45 (1), 55 (1), 85 (0), 98 (1). **Node 37**: 106 (0). **Node 38**: 18 (1), 22 (1), 28 (2), 53 (1), 54 (0), 66 (1). **Node 39**: 31 (1), 42 (1), 51 (1), 65 (1), 69 (1), 84 (0), 87 (2), 117 (1), 136 (0), 137 (1). **Node 40**: 56 (1), 88 (1). **Node 41**: 53 (1). **Node 42**: 61 (0). **Node 43**: 14 (1), 70 (0), 76 (1), 133 (1), 154 (1), 156 (1). **Node 44**: 16 (1), 20 (1), 21 (2), 22 (1), 23 (1), 27 (2), 78 (1), 80 (2), 114 (0), 120 (1).

Below, Bremer support (above) and bootstrap percentages (below) are given in each node:

**
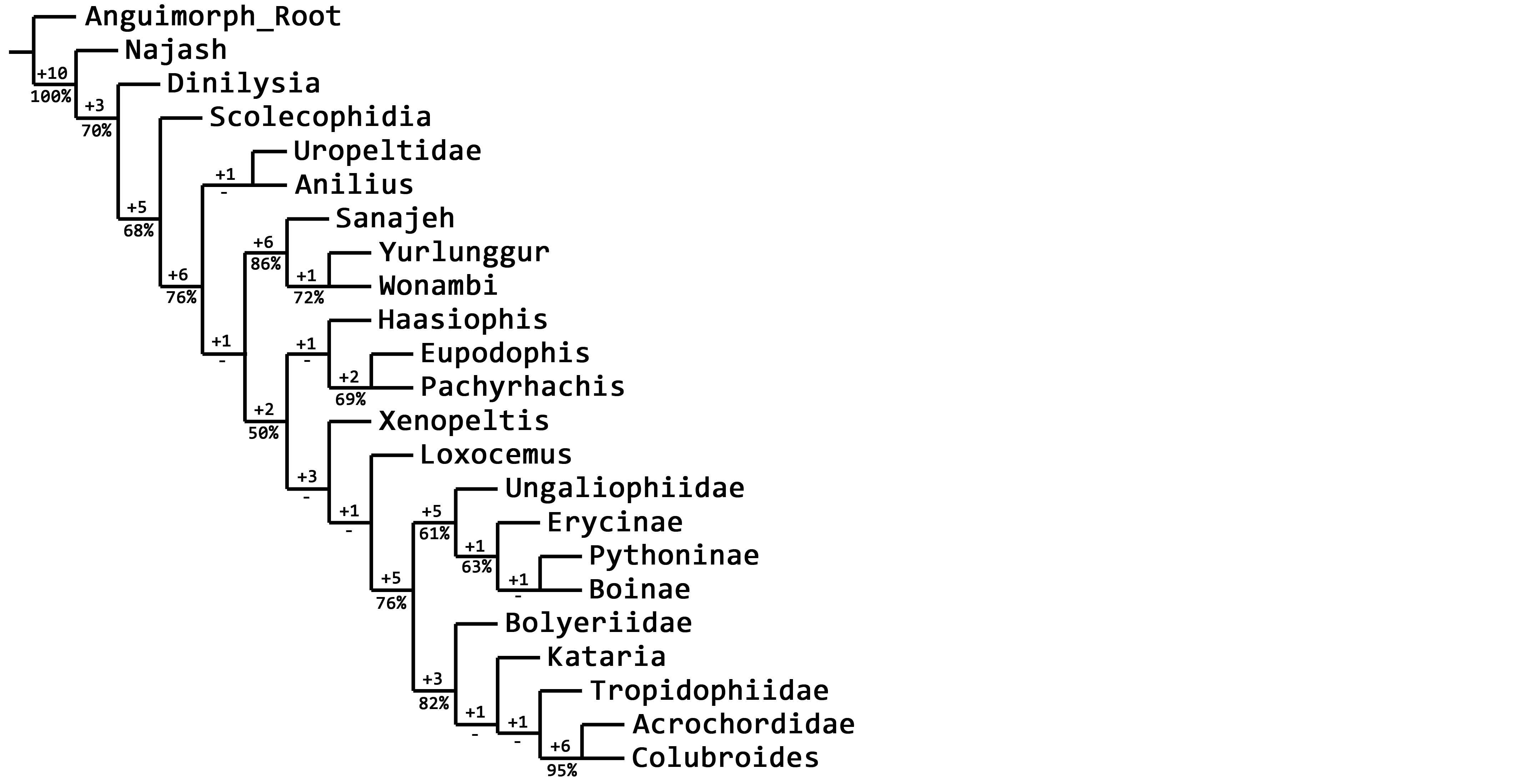
**

**1.4 Trees obtained in the constrained search using a molecular backbone phylogeny**

- Tree obtained by Wiens et al. [S16]


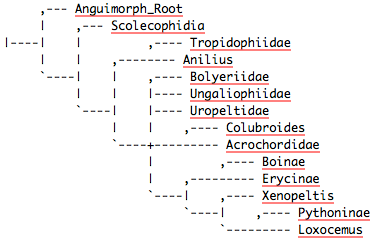


- Two most parsimonious trees recovered in the constrained analysis


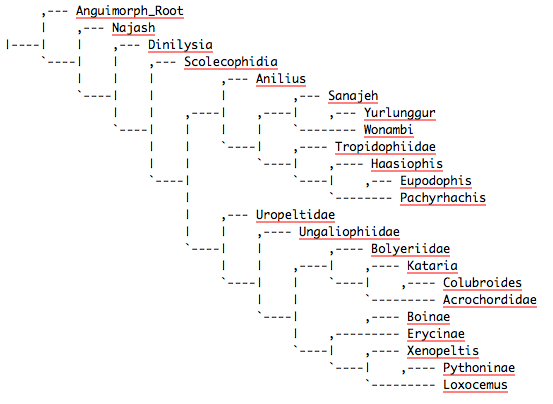


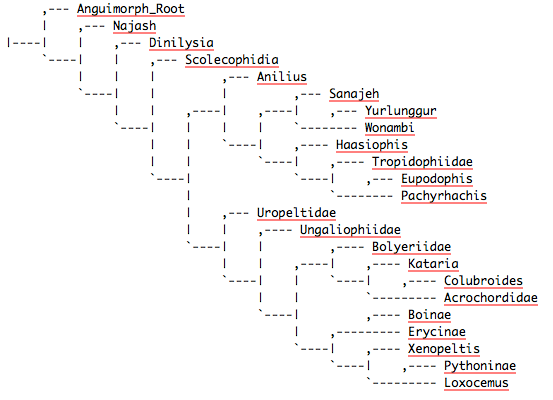


- Strict consensus tree of the two most parsimonious trees recovered in the constrained analysis


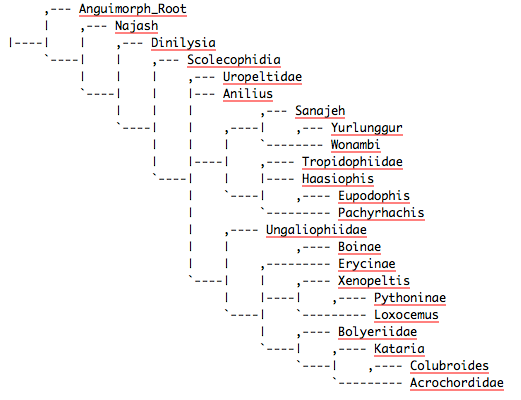


**2. Ages of appearance used in calibrated phylogeny**

We calibrated the obtained cladogram with the chronostratigraphic scale to know the existence and range of ghost lineages in the snake tree (Fig. 4). Also, we plotted first and last appearance of employed terminal taxa in the fossil record, discriminated in those records based on postcranial remains (usually not phylogenetically constrained) and records based on cranial/postcranial remains (usually phylogenetically constrained).

*Najash*: Upper Cretaceous (Cenomanian) – [S2]

*Dinilysia*: Upper Cretaceous (Santonian-Campanian) – [S17,S18]

Scolecophidia: Upper Cretaceous (Cenomanian) – [S19,S20,S21]

“Anilioid” clade: Lower-Upper Cretaceous (Albian-Cenomanian) – [S19,S20,S22,S23,S24,S25,S26,S27]

*Sanajeh*: Upper Cretaceous (Maastrichtian) – [S6]

*Yurlunggur*: Late Oligocene to Pleistocene – [S5]

*Wonambi*: Late Oligocene to Pleistocene – [S28]

*Haasiophis*: Upper Cretaceous (Cenomanian) – [S29]

*Eupodophis*: Upper Cretaceous (Cenomanian) – [S30,S31]

*Pachyrhachis*: Upper Cretaceous (Cenomanian) – [S32]

Erycinae: Late Oligocene – [S19,S20]

Boinae: Palaeocene – [S19,S20,S33,S34]

Pythoninae: Late Eocene – [S19,S20,S33,S35]

Acrochordidae: Early Miocene – [S36,S37]

Colubroides: Early-Middle Eocene – [S37,S38,S39,S40]

**3. Specimens examined**

Institutional abbreviations: AMNH, American Museum of Natural History; BMNH, British Museum of Natural History; CAS, California Academy of Science; CENAI, Centro Nacional de Investigaciones Biológicas (currently housed in MACN); CM, Carnegie Museum of Natural History; FML, Fundación Miguel Lillo; FMNH, Field Museum of Natural History; IAA, Instituto Antártico Argentino; IB, Instituto Butantan; LSUMZ, Louisiana State University Museum of Zoology; MACN, Museo Argentino de Ciencias Naturales “Bernardino Rivadavia”; MECN, Museo Ecuatoriano de Ciencias Naturales; MHNC, Museo de Historia Natural “Alcide D´Orbigny”; MLP, Museo de La Plata; MNHN, Museum National d´Histoire Naturelle; MPEG, Museu Paraense “Emílio Goeldi”; MPCA, Museo Provincial “Carlos Ameghino”; MUCPv, Museo Universidad Nacional del Comahue; MZUSP, Museu de Zoologia, Universidade de Sao Paulo; PVPH, Museo Carmen Funes.

*Achalinus formosanus* (LSUMZ 19354; BMNH 1983-192)*; Acrantophis madagascariensis* (MNHN 1983.484); *Acrantophis dumerili* (MZUSP 14430); *Acrochordus granulatus* (AMNH 66367); *Acrochordus javanicus* (AMNH 140813); *Ahaetulla ahaetulla* (MNHN C 952-36/-37/-38); *Ahaetulla prasina* (MNHN C 2943-29/-30/-31); *Ahaetulla subocularis* (MNHN 1973.142A); *Anilius scytale* (CENAI 3883, MACN 8817a, MACN 8817b, IB 46686, MZ 14572); *Antaresia childreni* (AMNH 86213); *Apostolepis erythronota* (AMNH 62192); *Apostolepis flavotorquata* (AMNH 93559); *Aspidites melanocephala* (AMNH 18681); *Atractaspis irregularis* (MNHN 1991.4071/4072); *Bitis arietans* (CENAI 3386); *Boa constrictor* (MACN 39025, MZUSP 2553, MZUSP 13843); *Boiruna maculata* (MACN 40006, MACN 40007); *Boiruna sertaneja* (MZUSP 7031); *Bungarus fasciatus* (CENAI 3887); *Calabaria reinhardtii* (AMNH 45901, CM 147738); *Candoia aspera* (AMNH 142843); *Candoia carinata* (MZ 14111, MZ 14112); *Casarea dussumieri* (MNHN 1992-27, MNHN 1993.3382); *Causus rhombeatus* (MNHN 1991.4146/4147); *Causus maculatus* (MNHN 1991.4140); *Causus resimus* (MNHN 1991.4144); *Cerberus rhynchops* (MNHN 1991.4352); *Charina bottae* (CM 36539, MZ, 8854); *Clelia rustica* (MACN 40004); *Corallus caninus* (CM 145320, IB 40869, MZUSP 14426); *Corallus hortulanus* (MZ 13050); *Cylindrophis maculatus* (AMNH 85496); *Cylindrophis ruffus* (AMNH 85647, CM 147774, MNHN 1970.411); *Daboia russelli* (MNHN 1991.4112/4113/4114, MNHN 1997.6005/6037); *Dasypeltis scabra* (CENAI 3853); *Dendroaspis polylepis* (MACN S/N); *Enhydris bocourti* (MNHN 1970-557A); *Enhydris enhydris* (MNHN C 3458-23/-24); *Enhydris innominata* (MNHN 1970-560A);  *Enhydris plumbea* (MNHN C 3461-15/-19); *Enhydris dussumieri* (MNHN 2009.0206); *Enhydris jagorii* (MNHN 1970.554A); *Enhydris sieboldii* (MNHN 2009.0204); *Epicrates angulifer* (CM 35999); *Epicrates cenchria* (IB 52174, 49335); *Erpeton tentaculatus* (MNHN 1970.573A); *Eryx conicus* (AMNH 89273, CM 91863); *Eryx jaculus* (MZ 14101); *Eryx johni* (AMNH 99701); *Eryx miliaris* (AMNH 143770); *Eunectes murinus* (MPEG 16443); *Eunectes notaeus* (MZUSP 7622); *Eunectes deschauensis* (MPEG 18019); *Exiliboa placata* (AMNH 102892); *Helicops leopardinus* (MACN 40014); *Homalopsis buccata* (MNHN 1991.4202, MNHN 1991.4347, MNHN 1991.4348, MNHN 1970.519a); *Homoroselaps lacteus* (MNHN 1991.4162); *Hydrophis* sp. (MNHN 1986.0596); *Langaha nasuta* (MNHN 1991.4355, MNHN 1950.178A); *Leiopython albertisii* (AMNH 95140, MZUSP 14427); *Liasis fuscus* (AMNH 86222); *Lichanura roseofusca* (CM 56093, CM 145332, MZUSP 7283); *Liophis anomalus* (MACN 40012); *Liophis miliaris* (MACN 40013); *Liophis poecilogyrus* (MACN 40011); *Loxocemus bicolor* (AMNH 110151, MZUSP 14114, FML 970, LSUMZ 49634); *Lystrophis dorbignyi* (MACN 40009); *Macrelaps microlepidotus* (CENAI 3858, LSUMZ 55387); *Madagascarophis colubrinus* (MNHN C 2451-37/-38); *Malpolon monsspesulanus* (MNHN 1988.6505, MNHN 1994.4175, MNHN 1991.4358, MNHN 1991.4562); *Mastigodryas bifossatus* (MACN 40017); *Mehelya capensis* (MACN 3857); *Mimophis madagascariensis* (MNHN 1989.2917, MNHN 1989.2918, MNHN 1989.2919, MNHN 1989.2961); *Morelia viridis* (AMNH 95135, MZUSP 14428); *Morelia spilota* (MNHN 1991.4048); *Naja nivea* (CENAI 3881); *Nerodia rhombifer* (CENAI 3838); *Notechis* sp. (MNHN 1991.4100); *Oxyrhabdium modestum* (LSUMZ 11814); *Oxyrhopus rhombifer* (MACN 40010); *Pareas mollendorfi* (AMNH 27770); *Parias sumatranus* (CENAI 3783); *Philodryas patagoniensis* (MACN 40008); *Philodryas mattogrossensis* (MACN 33420); *Philotamnus hoplogaster* (CENAI 3856); *Phimophis vittatus* (MACN 40005); *Psammophis crucifer* (MNHN 1991.4214); *Psammophis lineatus* (MNHN 1989.2942); *Psammophis sibilans* (MNHN 1991.4173, MNHN 1991.4565, MNHN 1991.4199/4200); *Pseudotyphlops philippinus* (BMNH 1978.1092); *Python molurus* (MACN 39026, MACN 39027); *Ramphiophis togoensis* (MNHN 1991.4184); *Ramphiophis maradiensis* (MNHN 1994.0587); *Rhinophis blythi* (AMNH 85076); *Rhinocerophis alternatus* (MACN 40018); *Rhinophis drummondhayi* (AMNH 85076); *Sanzinia madagascariensis* (MNHN 1900.122a); *Thamnodynastes hypoconia* (MACN 40016); *Trachyboa boulengeri* (MECN 2240, MZ 8247); *Trachyboa gularis* (AMNH 28982); *Tropidophis canus* (AMNH 73066, AMNH 45839); *Tropidophis feicki* (AMNH 81128, AMNH 81132); *Tropidophis melanurus* (AMNH 82880, AMNH 46690, AMNH 93002); *Tropidophis semicinctus* (AMNH 7386); *Tropidophis tackzanowskii* (MECN 3037); *Ungaliophis continentalis* (LSUMZ 55454); *Ungaliophis panamensis* (AMNH 58845, AMNH 62639); *Uropeltis brevis* (BMNH); *Uropeltis ceylonicus* (AMNH 43343); *Uropeltis pulmeyensis* (MNHN 1994-756); *Vipera aspis* (MNHN 1991.4066, MNHN 1991.4107, MNHN 1982.169A); *Vipera ammodytes* (MNHN 1991.4068); *Vipera berus* (MNHN 1991.4128); *Vipera latasti* (MNHN 1991.4145); *Waglerophis merremi* (MACN 40015); *Xenopeltis unicolor* (MACN 7568, MZUSP 9665, MNHN 1991.4446, FMNH 11524); *Xylophis perroteti* (MNHN 1991.4426).

Also, anatomical comparisons and some of the skull characters were codified trough the CT scan movies available from DigiMorph:

Maisano JA & O Rieppel. 2011. "*Casarea dussumieri*" (On-line), Digital

Morphology. Access August 2011 in http://digimorph.org/specimens/

Casarea_dussumieri.

The Deep Scaly Project. 2011. "*Boa constrictor*" (On-line), Digital Morphology. Access August 2011 in http://digimorph.org/specimens/Boa_constrictor.

The Deep Scaly Project. 2011. "*Xenopeltis unicolor*" (On-line), Digital

Morphology. Access August 2011 in http://digimorph.org/specimens/

Xenopeltis_unicolor.

The Deep Scaly Project. 2011. "*Eryx colubrinus*" (On-line), Digital Morphology.

Access August 2011 in http://digimorph.org/specimens/Eryx_colubrinus.

The Deep Scaly Project. 2011. "*Tropidophis haetianus*" (On-line), Digital

Morphology. Access August 2011 in http://digimorph.org/specimens/

**4. References cited in supplementary information**

1. Zaher, H. & Scanferla, A. 2012 The skull of the Upper Cretaceous snake *Dinilysia patagonica* Smith-Woodward, 1901, and its phylogenetic position revisited. *Zool. J. Linnean Soc.* 164, 194-238.
2. Apesteguía, S. & Zaher, H. 2006 A Cretaceous terrestrial snake with robust

Hindlimbs and a sacrum. *Nature* 440, 1037-1040.

1. Tchernov, E., Rieppel, O., Zaher, H., Polcyn, M. J. & Jacobs, I. J. 2000 A new fossil snake with limbs. *Science* 287, 2010–2012.
2. Rieppel O, Kluge AG, Zaher H. 2002. Testing the phylogenetic relationships of the Pleistocene snake *Wonambi naracoortensis* Smith. *Journal of Vertebrate Paleontology* 22: 812–829.
3. Scanlon, J. D. 2006 Skull of the large non-macrostomatan snake *Yurlunggur* from the Australian Oligo-Miocene. *Nature* 439, 839-842.
4. Wilson, J. A., Mohabey, D., Peters, S. & Head, J. J. 2010 Predation upon hatchling sauropod dinosaurs by a new basal snake from the Late Cretaceous of India. *PLoS Biol.* 8, 1-5.Scanlon, J. D. & Lee, M. S. Y. 2000 The Pleistocene serpent *Wonambi* and the early evolution of snakes. *Nature* 403, 416-420.
5. Zaher, H. & Rieppel, O. 1999 Tooth implantation and replacement in squamates, with special reference to mosasaur lizards and snakes. *Am. Mus. Novitat.* 3271, 1-19.
6. Kearney, M. & Rieppel, O. 2006 An investigation into the occurrence of plicidentine in the teeth of squamate reptiles. *Copeia* 2006 (3), 337-350.
7. Frazzetta, T. H. 1966 Studies on the morphology and function of the skull in the Boidae (Serpentes). Part 2. Morphology and function of the jaw apparatus in *Python sebae* and *Python molurus*. *J. Morphol.* 118, 217-296.
8. Zaher, H., Grazziotin, F. G., Cadle, J. E., Murphy, R. W., Moura-Leite, J. C. & Bonatto, S. L. 2009 Molecular phylogeny of advanced snakes (Serpentes, Caenophidia) with an emphasis on South American Xenodontines: a revised classification and descriptions of new taxa. *Pap. Avul. Zool.* 49 (11), 115-153.
9. Bogert, C. M. 1943 Dentitional phenomena in cobras and other elapids with

notes on adaptive modifications of fangs. *Bull. Am. Mus. Nat. His.* 81, 285–360.

1. Marx, H. & Rabb, G. B. 1972 Phyletic analysis of fifty characters of advanced

snakes. *Fieldiana: Zool.* 63, 1-321.

1. Cundall, D. & Irish, F. 2008 The snake skull. In *Biology of the Reptilia*, vol. 20, The skull of Lepidosauria (eds. C. Gans, A. S. Gaunt & K. Adler), pp. 349-692. Ithaca: New York Society for the Study of Amphibians and Reptiles.
2. Maddison, W. P. & Maddison, D. R. 2007 Mesquite: a modular system for

evolutionary analysis. Version 2.0 [http://mesquiteproject.org](http://mesquiteproject.org/)

1. Goloboff, P. A., Farris, J. S. & Nixon, K. 2008 TNT, a free program for phylogenetic analysis. *Cladistics* 24,774-786.
2. Wiens JJ, CR Hutter, DG Mulcahy, BP Noonan, TM Townsend, JW Sites Jr & TW Reeder. 2012. Resolving the phylogeny of lizards and snakes (Squamata) with extensive sampling of genes and species. *Biology Letters* 8 (6): 1043-1046.
3. Albino, A. 2007 Lepidosauromorpha. In *Patagonian Mesozoic Reptiles* (eds. Z. Gasparini, L. Salgado & R. A. Coria), pp. 87-115. Bloomington & Indianapolis: Indiana University Press.
4. Scanferla, A. & Canale, J. I. 2007 The youngest record of the Cretaceous snake genus *Dinilysia* (Squamata, Serpentes). *S. Am. J. Herpetol.* 2 (1), 76-81.
5. Rage, J-C. 1984 Serpentes. In *Handbuch der Paläoherpetologie* 11 (ed. P. Wellnhofer), pp. 1-80. Stuttgart: Gustav Fischer.
6. Rage, J-C. 1987 Fossil history. In *Snakes. Ecology and Evolutionary Biology* (eds. R. A. Seigel, J. T. Collins & S. S. Novak.), pp. 51-76. New York: Macmillan.
7. Augé, M. & Rage, J-C. 2006 Herpetofaunas from the Upper Paleocene and Lower Eocene of Morocco. *Ann. Paléont.* 92, 235–253.
8. Fox, R. C. 1975 Fossil snakes from the Upper Milk River Formation (Upper

Cretaceous), Alberta. *Can. J. Earth Sci.* 12, 1557-1563.

1. Armstrong-Ziegler, J. G. 1978 An aniliid snake and associated vertebrates from the Campanian of New Mexico. J. Paleon. 52, 480-483.
2. Gardner, J. D. & Cifelli, R. L. 1999 A primitive snake from the Cretaceous of

Utah. *Spec. Pap. Palaeont.* 60, 87-100.

1. Gómez, R. O., Báez, A. M. & Rougier, G. 2008 An anilioid snake from the Upper Cretaceous of northern Patagonia. *Cret. Res.* 29 (3), 481-488.
2. Bailon, S. 1988 Un aniliide´ (Reptilia, Serpentes) dans le Pliocène Supèrieur

Européen. *C. R. Acad. Sci.* 306 (2), 1255-1258.

1. Hsiou, A. S., Albino, A. M. & Ferigolo, J. 2010 Reappraisal of the South American Miocene snakes of the genus *Colombophis*, with description of a new species. *Acta Palaeont. Pol.* 55 (3), 365-379.
2. Scanlon, J. D. & Lee, M. S. Y. 2000 The Pleistocene serpent *Wonambi* and the early evolution of snakes. *Nature* 403, 416-420.
3. Rieppel, O., Zaher, H., Tchernov E. & Polcyn, M. J. 2003 The anatomy and relationships of *Haasiophis terrasanctus*, a fossil snake with well developed hind limbs from the mid-Cretaceous of the Middle East. *J. Paleont.* 77 (3), 536-558.
4. Rage, J-C. & Escuillié, F. 2000 Un nouveau serpent bipède du Cénomanien (Crétacé). Implications phylétiques. *C. R. Acad.* *Sci.* 330, 513-520.
5. Rieppel, O. & Head, J. J. 2004. New specimens of the fossil snake genus *Eupodophis* Rage & Escuillié, from Cenomanian (Late Cretaceous) of Lebanon. *Mem. Soc. Ital. Sci. Nat. Mus Civ. Stor. Nat. Milano* 32 (2), 1-26.
6. Lee, M. S. Y. & Caldwell, M. W. 1998 Anatomy and relationships of *Pachyrhachis problematicus*, a primitive snake with limbs. *Phil. Trans. Roy. Soc. London* B 353, 1521-1552.
7. Szyndlar, Z & Rage J-C. 2003 *Non-erycine Booidea from the Oligocene and*

*Miocene of Europe*. Institute of Systematics and Evolution of Animals,

Polish Academy of Sciences, Kraków, 109 pp.

1. Head, J. J., Bloch, J. I., Hastings, A. K., Bourque, J. R., Cadena, E. A., Herrera, F. A., Polly, P. D. & Jaramillo, C. A. 2009 Giant boid snake from the Palaeocene neotropics reveals hotter past equatorial temperatures. *Nature* 457, 715-718.
2. Scanlon, J. D. 2001 *Montypythonoides*: the Miocene snake *Morelia riversleighensis* (Smith & Plane, 1985) and the geographical origin of pythons. *Mem. Assoc. Austr. Palaeont.* 25, 1-35.
3. Head, J. J. 2005 Snakes of the Siwalik group (Miocene of Pakistan): systematics and relationship to environmental change. *Palaeont. Elect.* 8 (1), 1-33.
4. Head, J. J., Holroyd, P. A., Hutchison, J. H. & Ciochon, R. L. 2005 First report of snakes (Serpentes) from the late middle Eocene Pondaung Formation, Myanmar. *J. Vert. Paleont.* 25, 246–250.
5. Parmley, D. & Holman, J. A. 2003 *Nebraskophis* Holman from the Late Eocene of Georgia (USA), the oldest known North American colubrid snake. *Acta Zool. Cracov.* 46 (1), 1-8.
6. Rage, J-C, Buffetaut, E., Buffetaut-Tong, H., Chaimanee, Y., Ducrocq, S., Jaeger

J-J. & Suteethorn, V. 1992 A colubrid snake in the late Eocene of Thailand:

the oldest known Colubridae (Reptilia, Serpentes). *C. R. Acad. Sci.*

2 (314), 1085-1089.

1. Rage, J-C., Folie, A., Rana, R. S., Singh, H., Rose, K. D. & Smith, T. 2008 A diverse snake fauna from the early Eocene of Vastan Lignite Mine, Gujarat, India. *Acta Palaeont. Pol.* 53 (3), 391-403.
